# Youthful Stem Cell Microenvironments: Rejuvenating Aged Bone Repair Through Mitochondrial Homeostasis Remodeling

**DOI:** 10.1002/advs.202409644

**Published:** 2025-01-17

**Authors:** Xinfeng Zhou, Xin Tian, Jianan Chen, Yantong Li, Nanning Lv, Hao Liu, Tao Liu, Huilin Yang, Xi Chen, Yong Xu, Fan He

**Affiliations:** ^1^ Department of Orthopaedics The First Affiliated Hospital of Soochow University Orthopedic Institute MOE Key Laboratory of Geriatric Diseases and Immunology Suzhou Medical College Soochow University Suzhou Jiangsu 215000 China; ^2^ Department of Orthopaedics The Third Affiliated Hospital of Soochow University Changzhou Jiangsu 213000 China; ^3^ Department of Pathology The Third Affiliated Hospital of Soochow University Changzhou Jiangsu 213000 China

**Keywords:** aged bone defects, bone marrow‐derived mesenchymal stem cells, mitochondrial energy metabolism, SIRT3, youthful extracellular matrix

## Abstract

Extracellular matrix (ECM) derived from mesenchymal stem cells regulates antioxidant properties and bone metabolism by providing a favorable extracellular microenvironment. However, its functional role and molecular mechanism in mitochondrial function regulation and aged bone regeneration remain insufficiently elucidated. This proteomic analysis has revealed a greater abundance of proteins supporting mitochondrial function in the young ECM (Y‐ECM) secreted by young bone marrow‐derived mesenchymal stem cells (BMMSCs) compared to the aged ECM (A‐ECM). Further studies demonstrate that Y‐ECM significantly rejuvenates mitochondrial energy metabolism in adult BMMSCs (A‐BMMSCs) through the promotion of mitochondrial respiratory functions and amelioration of oxidative stress. A‐BMMSCs cultured on Y‐ECM exhibited enhanced multi‐lineage differentiation potentials in vitro and ectopic bone formation in vivo. Mechanistically, silencing of silent information regulator type 3 (SIRT3) gene abolished the protective impact of Y‐ECM on A‐BMMSCs. Notably, a novel composite biomaterial combining hyaluronic acid methacrylate hydrogel microspheres with Y‐ECM is developed, which yielded substantial improvements in the healing of bone defects in an aged rat model. Collectively, these findings underscore the pivotal role of Y‐ECM in maintaining mitochondrial redox homeostasis and present a promising therapeutic strategy for the repair of aged bone defects.

## Graphical abstract

This study introduces a novel bone regeneration platform combining young ECM (Y‐ECM) with hydrogel microspheres, which remodels mitochondrial homeostasis in stem cells. In vivo, HM@Y‐ECM exhibits a rejuvenating effect on aged bone defects similar to “dead wood coming to life in spring”. It may lead to the development of biomaterials‐based therapeutics avoiding some intrinsic complications associated with cell‐based therapeutics.

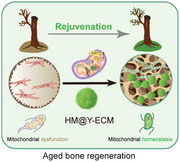

## 1 Introduction

With the global rise in the aging population, repairing bone defects in aged people has become a significant challenge for public healthcare systems. The destruction of bone homeostasis and the decline of regenerative potential caused by stem cell senescence are considered to be one of the main reasons for the difficulty in aged bone regeneration.^[^ ^1^ ^]^ The increased occurrence of cellular senescence plays a pivotal role in the onset of aging and associated health issues.^[^ ^2^ ^]^ The characteristic of cellular senescence is the persistent arrest of the cell cycle, during which the cells acquire a senescence‐associated secretory phenotype (SASP). This senescent microenvironment drives tissue dysfunction in a negative feedback loop, thereby significantly hindering tissue regeneration in the elderly.^[^ ^3^ ^]^ Bone marrow‐derived mesenchymal stem cells (BMMSCs) are the primary source of bone‐forming cells, i.e., osteoblasts, but they undergo senescence in naturally aged bone. Senescent BMMSCs not only exhibit diminished capacities in self‐renewal and differentiation, but also secrete SASP molecules that potentiate inflammatory responses and exacerbate bone degradation. It is worth noting that mitochondrial dysfunction is closely associated with cellular senescence and is considered as one of the key factors that affect bone regeneration in recent years.^[^ ^4^ ^]^ Most likely, the accumulation of dysfunctional mitochondria in senescent BMMSCs over time results in a surge of reactive oxygen species (ROS), an upsurge in inflammation, and a disruption in energy metabolism, thereby contributing to the disturbance of bone homeostasis.^[^ ^1^, ^5^ ^]^ Therefore, exploring novel materials to restore the mitochondrial function of BMMSCs may lead to the discovery of new therapeutic strategies to promote the regeneration of aged bone defects.

Currently, several rejuvenation strategies targeting mitochondrial functions have been reported, such as the use of small molecule drugs, genetic reprogramming, and extracellular matrix (ECM).^[^ ^6^ ^]^ In these anti‐aging strategies, MSC‐derived ECM (mECM) has attracted increasing attention from researchers due to its high degree of simulation of the natural stem cell microenvironment. The ECM is a fundamental component of tissue microenvironment, providing soluble and insoluble cues that guide cell adhesion, migration, proliferation, and differentiation.^[^ ^7^ ^]^ Previous studies have shown that mECM not only promotes the synthesis of bone matrix but also regulates osteoclast‐mediated bone resorption, playing a key role in the regulation of bone metabolism.^[^ ^8^ ^]^ More importantly, mECM can enhance the antioxidant function of MSCs by increasing the expression and activity of antioxidant enzymes such as superoxide dismutases (SODs) and catalase (CAT).^[^ ^9^ ^]^ Since mitochondrial dysfunction is closely related to oxidative stress, mECM has been found to significantly improve cellular adenosine triphosphate (ATP) production without any toxic side effects, demonstrating excellent potential for mitochondrial protection.^[^ ^10^ ^]^ Pioneering research by Conboy et al. demonstrated that a youthful microenvironment effectively promotes tissue regeneration, whereas an aged microenvironment fails to support this process.^[^ ^11^ ^]^ Subsequent studies further elucidated that the youthful microenvironment, especially the ECM, is pivotal in determining the quality and quantity of MSCs. MSCs isolated from elderly donors show diminished proliferation capacity and osteogenic potential in vitro; however, when these cells were cultured on ECM derived from young donors (Y‐ECM), their osteogenic potential was markedly enhanced.^[^ ^12^ ^]^ Considering the key role of mitochondria in the rejuvenation process, we hypothesize that Y‐ECM can promote the regeneration of aged bone tissues by remodeling the mitochondrial homeostasis in BMMSCs. However, the specific effects of Y‐ECM on BMMSCs’ mitochondrial functions and the key molecular mechanisms need to be fully elucidated.

The Sirtuin (SIRT) family, highly conserved NAD^+^‐dependent enzymes, is increasingly recognized as a key regulator of stem cell biology.^[^ ^13^ ^]^ A previous study from our laboratory revealed that activation of SIRT1 not only suppresses the expression of the senescence‐associated protein P16^INK4α^ in MSCs, but also enhances their mitochondrial energy metabolism and the osteogenic potential.^[^ ^14^ ^]^ However, given that SIRT1 is predominantly nuclear‐localized, its regulatory influence on mitochondrial function is primarily exerted through indirect mechanisms. For example, SIRT1 modulates mitochondrial biogenesis and energy metabolism by deacetylating the mitochondrial protein peroxisome proliferator‐activated Receptor gamma coactivator 1‐alpha (PGC‐1α).^[^ ^15^ ^]^ It is noteworthy that SIRT3, a deacetylase located in the mitochondria, plays an essential role in maintaining mitochondrial homeostasis, enhancing antioxidant defense, preserving genome stability, and epigenetic regulation.^[^ ^16^ ^]^ Diao et al. found that the expression of SIRT3 was decreased in senescent human MSCs, while knockout of SIRT3 resulted in impaired nuclear integrity, loss of heterochromatin, and accelerated cellular senescence.^[^ ^17^ ^]^ Furthermore, the SIRT3/SOD2 axis has been demonstrated to play a crucial role in osteoblast differentiation and bone formation through the regulation of mitochondrial redox homeostasis.^[^ ^18^ ^]^ Intriguingly, the decreased SIRT3 in elderly mice is associated with the degeneration of osteocyte function, the decline in bone mass, and a reduction in the bone‐enhancing effects of exercise, suggesting the protective effect of SIRT3 on age‐related bone defect.^[^ ^19^ ^]^ However, the precise role of SIRT3 in promoting the healing of aged bone defects is yet to be determined.

Hyaluronic acid (HA), a natural glycosaminoglycan, is renowned for its biocompatibility as a template for biomineralization, making it an ideal candidate for bone regeneration.^[^ ^20^ ^]^ However, HA alone is insufficient for bone regeneration due to the lack of osteoinduction.^[^ ^21^ ^]^ It is worth highlighting that hydrogel microspheres (HMs) exhibit pronounced benefits compared to conventional bulk hydrogels with respect to mechanical performance, microporous structure, and injectability.^[^ ^22^ ^]^ Despite its potent biological activity, the mECM, derived from decellularized cultures of highly confluent BMMSCs in vitro, lacks mechanical strength and important glycosaminoglycan components.^[^ ^23^ ^]^ Therefore, in this study we will test whether Y‐ECM can provide a comprehensive rejuvenating extracellular microenvironment for adult BMMSCs (A‐BMMSCs), thereby remodeling their mitochondrial homeostasis and promoting the repair of aged bone defects. Mechanistically, we will investigate the role of SIRT3 in Y‐ECM‐mediated protection on mitochondrial functions. Finally, to evaluate its clinical application potential, we will develop a composite microsphere combining methacrylated HA (HAMA) and Y‐ECM and investigate the effect of the HM@Y‐ECM microspheres on vascularized bone regeneration by *in‐situ* implantation in aged rats’ calvarial defects.

## 2 Results

### 2.1 A‐ECM and Y‐ECM Display Substantial Differences in Microstructure, Mechanical Properties, and Protein Composition

**Figure 1:**
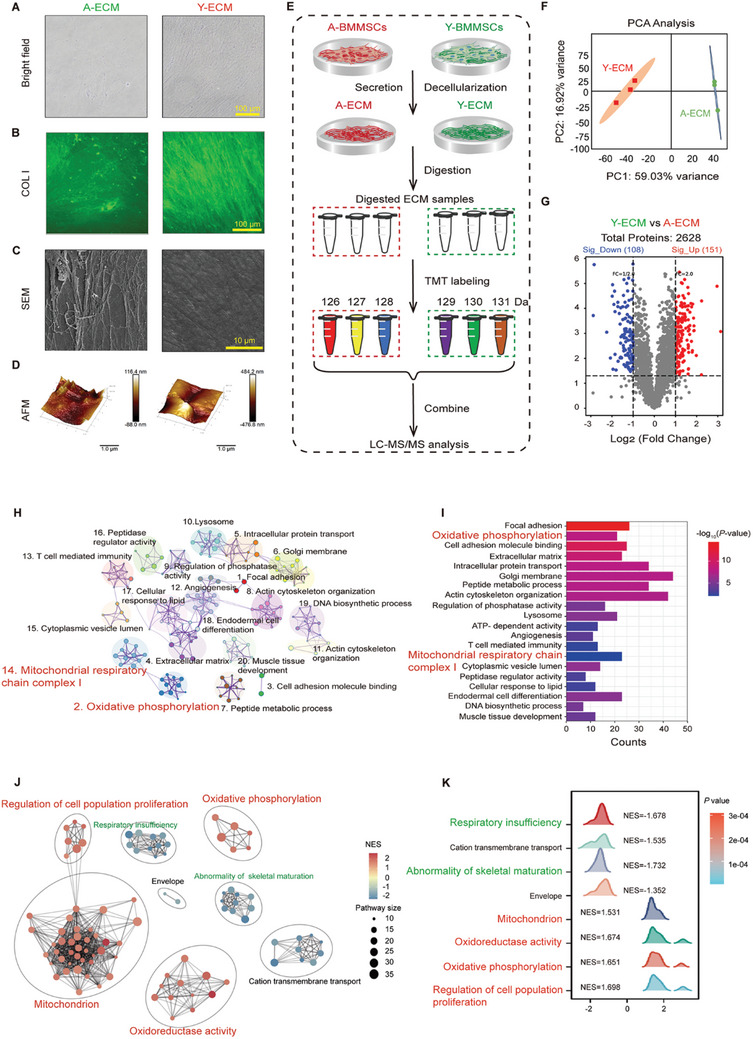
Comparison of matrix microstructure and component composition in A‐ECM and Y‐ECM. A) Optical microscope for gross structure. B) Immunostaining for type I collagen (COL I). C) SEM for the surface microstructure of A‐ECM and Y‐ECM. D) AFM for surface morphology. E) TMT labeling experimental workflow. F) PCA plot of proteomic data in different groups. G) Volcano plots of protein expression levels in Y‐ECM versus A‐ECM. H) Network of enriched pathways of differentially expressed proteins in Y‐ECM versus A‐ECM. Each node and its size represent a statistically enriched pathway and the number of enriched proteins on each node pathway, respectively. The clusters of similar paths are marked with the same color. I) Bar charts plot the cluster significance of enrichment pathways of differentially expressed proteins in Y‐ECM versus A‐ECM. J) Enrichment network based on GSEA database resources. The nodes represent the significant pathways and the edges represent similarity between them. Colored by normalized enrichment score (NES). K) Ridge plot based on GSEA database resources.

The ECM constitutes a crucial component of the extracellular microenvironment and exerts significant regulatory effects on the cellular behaviors of MSCs. Light microscopy showed that the fiber network structure of Y‐ECM exhibited a higher density compared to that of A‐ECM. (**Figure** **1**A). From immunofluorescence staining, we identified COL I, one of the major protein components of ECM, in both A‐ECM and Y‐ECM (Figure 1B). SEM was used to characterize the microstructure of Y‐ECM and A‐ECM (Figure 1C). The surface of Y‐ECM appeared smoother and more uniform compared to the rough surface of A‐ECM, indicating a higher level of uniformity and slenderness in the matrix fibers of Y‐ECM. In accordance with the SEM results, AFM revealed that the surface of A‐ECM exhibited a rough and irregular topography. Conversely, Y‐ECM displayed a smoother surface morphology accompanied by a denser matrix structure (Figure 1D). To further determine the mechanical difference between A‐ECM and Y‐ECM, AFM results showed that the average stiffness (elastic modulus) of Y‐ECM was significantly lower than that of A‐ECM (Figure S1A, Supporting Information).

To comprehensively investigate the compositional disparities between A‐ECM and Y‐ECM, we employed a standardized workflow to extract ECM proteins and conducted a TMT‐based quantitative proteome analysis (Figure 1E). The principal component analysis (PCA) for the ECM revealed that each group was assembled into a clear cluster, indicating a high level of replicates consistency within the respective groups (Figure 1F). Notably, we identified 259 differentially expressed proteins (DEPs) (*p* < 0.05) in Y‐ECM versus A‐ECM, with 151 up‐regulated and 108 down‐regulated proteins (Figure 1G). As shown in Figure S1B (Supporting Information), several proteins of interest were identified, whose functions are involved in mitochondrial homeostasis as well as cell senescence. We assumed that DEPs may lead to distinct biological functionalities in Y‐ECM compared to A‐ECM. Subsequently, we conducted pathway enrichment analysis on DEPs using Metscape. The results demonstrated significant enrichment of several biological processes, including “oxidative phosphorylation” and “mitochondrial respiratory chain complex I”(Figure 1H,I). In addition, to address the limitations of DEPs analysis, GSEA results revealed that the up‐regulated proteins in Y‐ECM were involved in the regulation of mitochondrion, oxidoreductase activity, oxidative phosphorylation, and cell proliferation, while the down‐regulated proteins in Y‐ECM were associated with respiratory insufficiency and abnormality of skeletal maturation (Figure 1J,K). These results suggest that Y‐ECM may exert a beneficial effect on cellular mitochondrial energy metabolism.

### 2.2 Y‐ECM Rejuvenated A‐BMMSCs by Remodeling Mitochondrial Homeostasis

We first compared the differences in biological functions between F‐BMMSCs and A‐BMMSCs. Experimental data showed that the self‐renewal capacity (Figure S2, Supporting Information), multi‐lineage differentiation potentials (Figure S3, Supporting Information), and antioxidant capacity (Figure S4, Supporting Information) in A‐BMMSCs were remarkably lower than F‐BMMSCs. TEM revealed that mitochondria in A‐BMMSCs exhibited swollen and degenerated in contrast to F‐BMMSCs’ mitochondrion morphology (Figure S5A, Supporting Information). In terms of mitochondrial energy metabolism, both ATP production (Figure S5B, Supporting Information) and MMP levels (Figure S5C, Supporting Information) were significantly reduced in A‐BMMSCs. Regarding mitochondrial biosynthesis, the mtDNA copies (Figure S5D, Supporting Information) and the expression of mitochondrial respiratory chain factors (ND4, SDHA, UQCRC1, COX4, and ATP5A) (Figure S5E–G, Supporting Information) exhibited a significant decrease in A‐BMMSCs.

**Figure 2:**
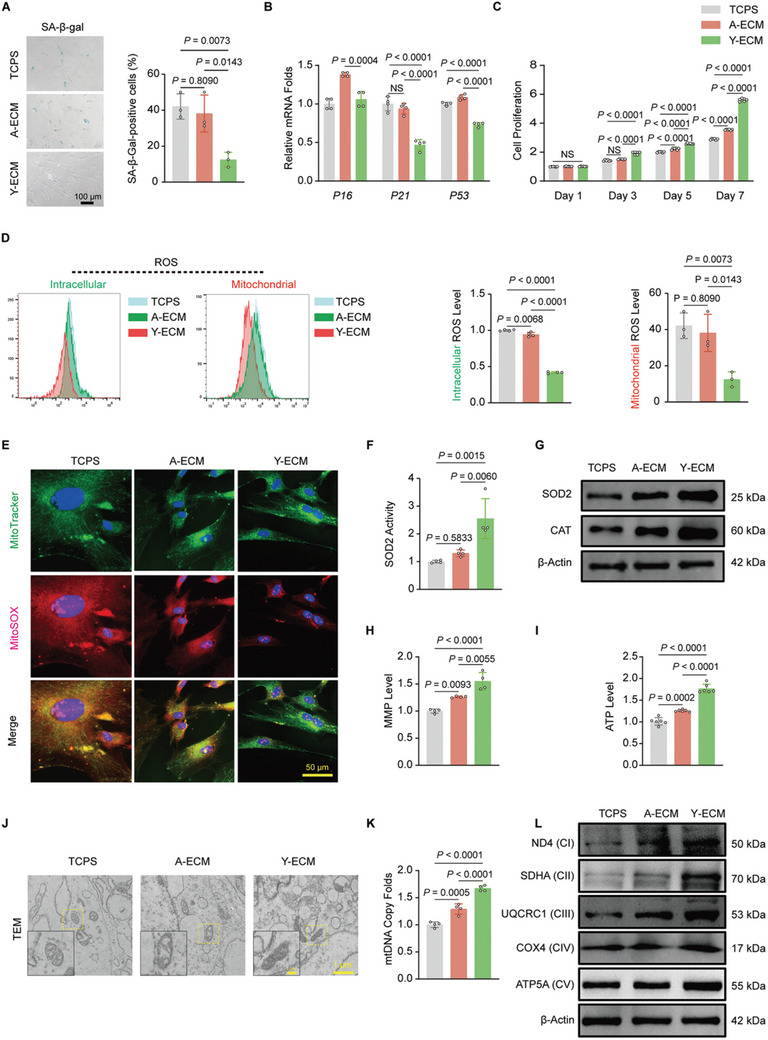
Y‐ECM prevented cellular senescence and improved mitochondrial functions of A‐BMMSCs. A) Representative SA‐β‐gal staining images and quantification of expanded A‐BMMSCs (n = 3 for each group, one‐way ANOVA). B) The mRNA expression levels of senescence‐related genes (*P16*, *P21*, *P53*) were quantified using RT‐qPCR (n = 4 for each group, one‐way ANOVA). C) CCK‐8 assay was used to measure the proliferation of expanded A‐BMMSCs (n = 6 for each group, one‐way ANOVA). D) Y‐ECM effectively reduced intracellular and mitochondrial ROS levels (n = 4 for each group, one‐way ANOVA). E) Mitochondrial ROS was evaluated using MitoTracker (green) and MitoSOX (red) double‐staining. F) Y‐ECM improved SOD2 activity of A‐BMMSCs (n = 4 for each group, one‐way ANOVA). G) Y‐ECM effectively increased the protein levels of SOD2 and CAT (n = 3 for each group, one‐way ANOVA). H, I) Y‐ECM effectively increased ATP production (n = 6 for each group, one‐way ANOVA) and MMP levels (n = 4 for each group, one‐way ANOVA) of expanded A‐BMMSCs. J) Representative TEM images of mitochondria in expanded A‐BMMSCs. K) Measurement of mtDNA copy number in expanded A‐BMMSCs (n = 4 for each group, one‐way ANOVA). L) Protein levels of ND4, SDHA, UQCRC1, COX4, and ATP5A (n = 3 for each group, one‐way ANOVA). Data are presented as mean ± SD of at least three independent assays for each experiment. Statistically significant differences between groups are set at *p* < 0.05.

To investigate the effects of A‐ECM and Y‐ECM on cell behaviors, A‐BMMSCs were seeded onto TCPS, A‐ECM, and Y‐ECM substrates, respectively. TCPS‐ or A‐ECM‐cultured A‐BMMSCs appeared flattened and enlarged morphology, whereas Y‐ECM‐cultured cells exhibited a significantly smaller size and a fibroblast‐like shape (Figure S6, Supporting Information). Subsequently, we observed that Y‐ECM‐cultured A‐BMMSCs demonstrated a significant reduction in SA‐β‐gal activity (**Figure** **2**A), indicating that Y‐ECM suppressed the cellular senescence. The results of SA‐β‐gal staining were further supported by RT‐qPCR, evidenced by a significant decrease in mRNA expression of *P21* (by 53.3%) and *P53* (by 26.7%) in A‐BMMSCs, compared to the TCPS group (Figure 2B). Furthermore, the Y‐ECM group had the highest rate of cell proliferation, followed by the A‐ECM group, while the TCPS group exhibited the lowest rate (Figure 2C).

The ROS level in expanded A‐BMMSCs was effectively reduced by both A‐ECM and Y‐ECM. Interestingly, the ROS scavenging ability of Y‐ECM was superior to that of A‐ECM. The Y‐ECM treatment significantly reduced intracellular and mitochondrial ROS levels, with decreases of 46.4% and 55.7%, respectively, compared to the A‐ECM group (Figure 2D). The immunofluorescence staining results also demonstrated the effective mitigation of mitochondrial ROS levels following Y‐ECM treatment (Figure 2E). Furthermore, Y‐ECM improved SOD2 activity in A‐BMMSCs (Figure 2F). Significantly, the gene expression levels of *SOD2* and *CAT* in the Y‐ECM group were 5.2 and 1.1‐fold higher than those in the A‐ECM group, respectively (Figure S7B, Supporting Information). Western blot analysis showed that, compared with the TCPS group, Y‐ECM significantly increased the protein levels of SOD2 and CAT by 1.7 and 1.2‐fold, respectively, in A‐BMMSCs (Figure 2G; Figure S7C, Supporting Information**)**.

The rejuvenation capacity of Y‐ECM was further examined to evaluate its influence on mitochondrial function in A‐BMMSCs. Impressively, Y‐ECM significantly enhanced the mitochondrial energy metabolism in A‐BMMSCs, as evidenced by a remarkable 55.0% increase in MMP and a substantial 74.5% enhancement in ATP production compared to the TCPS group (Figure 2H,I). Additionally, TEM analysis results revealed that only Y‐ECM‐cultured A‐BMMSCs exhibited significant alterations in mitochondrial morphology, characterized by a decrease in swollen shape and restoration of cristae structure. Conversely, disparity was barely observed in mitochondrial morphology in the TCPS and A‐ECM groups (Figure 2J). It is noteworthy that following expansion on Y‐ECM, mitochondrial biosynthesis of A‐BMMSCs was significantly enhanced compared to the TCPS group. This enhancement was evidenced by a 66.9% increase in mtDNA copy number (Figure 2K) and substantial up‐regulation of mitochondrial respiratory chain factors, e.g., ND4, SDHA, UQCRC1, COX4, and ATP5A (Figure 2L; Figure S8, Supporting Information).

### 2.3 Y‐ECM Improved the Multi‐Lineage Differentiation Potential of A‐BMMSCs in vitro and Promoted Ectopic Bone Formation in vivo

**Figure 3:**
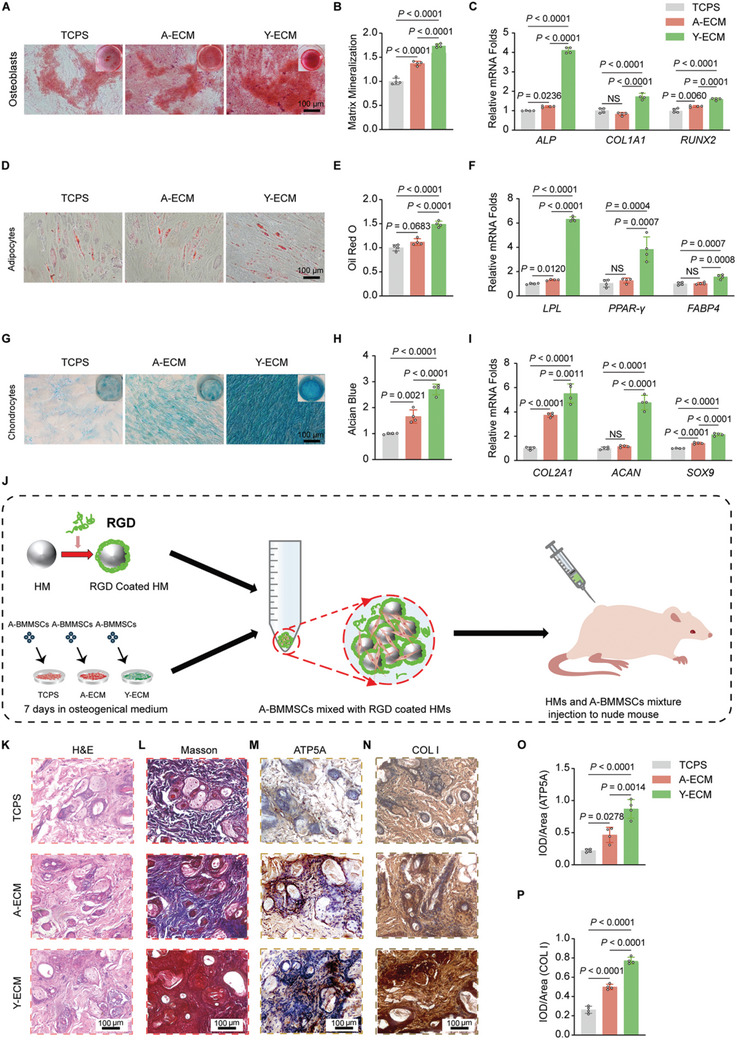
Y‐ECM improved the multi‐lineage differentiation potentials of A‐BMMSCs. A,B) Representative images and quantitative analysis of ARS staining on day 21 were conducted to evaluate matrix mineralization (n = 4 for each group, one‐way ANOVA). C) Real‐time PCR was used to evaluate osteoblast‐specific gene expression (*ALP*, *COL1A1*, and *RUNX2*) on day 21 (n = 4 for each group, one‐way ANOVA). D,E) Representative images and quantitative analysis of Oil red O staining on day 21 were conducted to examine lipid formation in mature adipocytes (n = 4 for each group, one‐way ANOVA). F) Real‐time PCR was used to evaluate adipocyte‐specific gene expression (*LPL*, *PPAR‐γ*, and *FABP4*) on day 21 (n = 4 for each group, one‐way ANOVA). G,H) Representative images and quantitative analysis of Alcian blue staining on day 21 were conducted to evaluate the content of GAGs (n = 4 for each group, one‐way ANOVA). I) Real‐time PCR was used to evaluate chondrocyte‐specific gene expression (*COL2A1*, *ACAN*, and *SOX9*) on day 21 (n = 4 for each group, one‐way ANOVA). J) Schematic illustration of subcutaneous implantation of HAMA microspheres loaded with A‐BMMSCs in nude mice. K–N) Representative histological and immumohistochemical images of grafts at 2 months after being subcutaneously injected into the nude mice back: K) H&E staining, L) Masson's trichrome staining, M) ATP5A staining, N) COL I staining. O,P) Quantitative analysis for ATP5A and COL I (n = 4 for each group, one‐way ANOVA). Data are presented as mean ± SD of at least four independent assays for each experiment. Statistically significant differences between groups are set at *p* < 0.05.

To investigate the regenerative effects of A‐ECM and Y‐ECM on the multi‐lineage differentiation potentials of A‐BMMSCs in vitro, expanded A‐BMMSCs were induced toward osteogenesis, adipogenesis, and chondrogenesis, respectively. The ARS staining results demonstrated that A‐BMMSCs expanded on the three different substrates were able to induce matrix mineralization (**Figure** **3**A). Notably, the Y‐ECM group displayed the highest level of calcium deposition and expression of osteoblast‐specific marker genes such as *ALP*, *COL1A1*, and *RUNX2* (Figure 3B,C). Consistent with the trend of osteogenic differentiation, we observed a significant enhancement in the adipogenic differentiation of Y‐ECM‐expanded A‐BMMSCs, as evidenced by higher levels of mature lipids that were positive for Oil Red O staining (Figure 3D,E). Accordingly, Y‐ECM substantially up‐regulated the expression levels of adipocyte‐specific markers including *LPL*, *PPAR‐γ*, and *FABP4* (Figure 3F). With regard to chondrogenesis, Y‐ECM significantly enhanced the production of sulfated GAGs in A‐BMMSCs (Figure 3G,H). The expression of chondrocyte‐specific genes *COL2A1*, *ACAN*, and *SOX9* was also significantly up‐regulated in the Y‐ECM group (Figure 3I).

We subsequently assessed the impact of pretreatment with A‐ECM and Y‐ECM on the osteogenic differentiation activity of A‐BMMSCs in vivo, utilizing a nude mouse ectopic bone formation model. A‐BMMSCs were cultured on TCPS, A‐ECM, and Y‐ECM for 7 days to induce osteogenesis in vitro. Following the induction, the three groups of A‐BMMSCs were loaded onto RGD‐modified HAMA microspheres and injected subcutaneously into the back of immunodeficient mice (Figure 3J). At 8 weeks post‐implantation, the H&E staining demonstrated that Y‐ECM‐cultured A‐BMMSCs exhibited enhanced adherence to the surrounding tissues in vivo compared to the other two groups (Figure 3K). The Masson's trichrome staining results revealed a sparse distribution of blue collagen fibers surrounding the microspheres in the TCPS group. In contrast, the A‐ECM group exhibited a significant increase in blue collagen fibers and the presence of newly formed bone tissues. Notably, the Y‐ECM group displayed an abundance of interconnected calcified bone‐like tissues that were stained dark red, indicating a higher degree of bone maturation compared to other two groups (Figure 3L). The immunohistochemical staining results revealed that the Y‐ECM group exhibited the highest intensity of ATP5A protein expression compared to the other two groups, indicating that the Y‐ECM pretreatment significantly enhanced mitochondrial function of A‐BMMSCs (Figure 3M,O). The results of COL I staining further confirmed that the Y‐ECM pretreatment significantly enhanced in vivo bone formation of A‐BMMSCs compared to the other two groups (Figure 3N,P).

### 2.4 Y‐ECM Elevated SIRT3 Levels in A‐BMMSCs to Improve Mitochondrial Energy Metabolism

**Figure 4:**
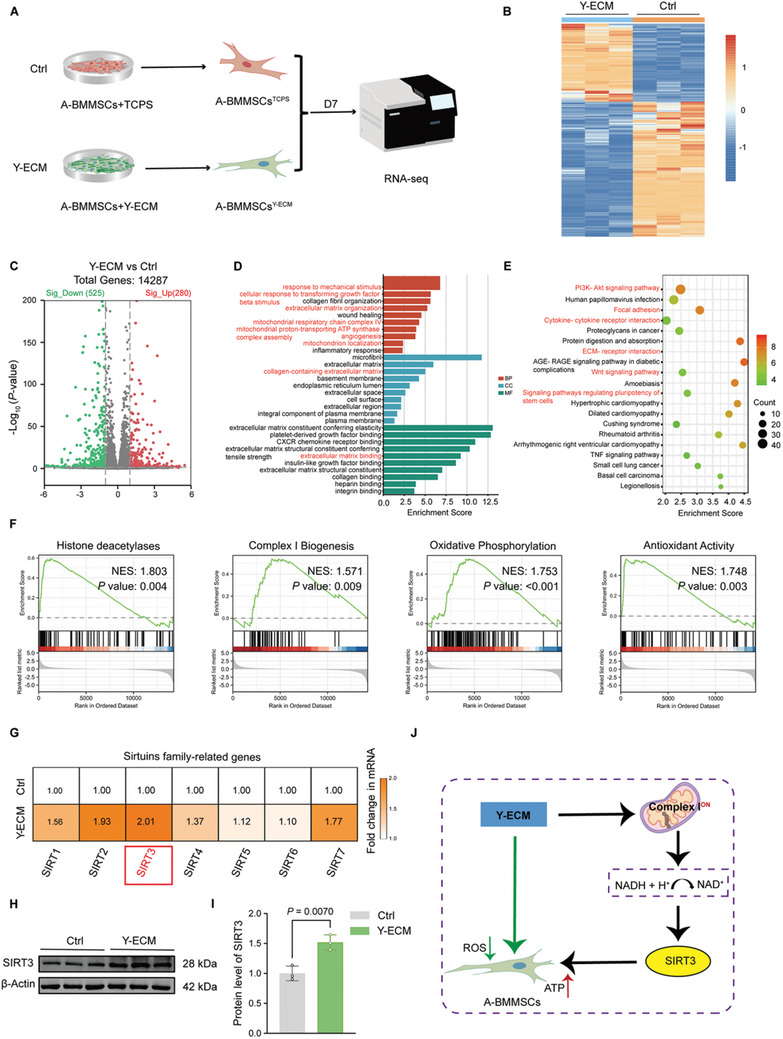
Elevated SIRT3 levels were detected in A‐BMMSCs during in vitro expansion on Y‐ECM. A) Schematic of the transcriptomic profiling of A‐BMMSCs expanded on Y‐ECM or TCPS (Ctrl). B) Heat maps of DEGs between A‐BMMSCs and Y‐ECM‐treated A‐BMMSCs. C) Volcano plots of gene expression levels in Y‐ECM versus Ctrl. D) Top GO terms significantly enriched from DEGs. E) Top KEGG pathways significantly enriched from DEGs. F) GSEA analysis of genes associated with histone deacetylases, complex I biogenesis, oxidative phosphorylation, and antioxidant activity. G) RT‐qPCR of Sirtuins family‐related gene expression (SIRT1‐7) (n = 4 for each group, unpaired two‐tailed Student's *t*‐test). H,I) Y‐ECM promoted the SIRT3 protein expression in A‐BMMSCs (n = 3 for each group, unpaired two‐tailed Student's *t*‐test). J) Y‐ECM promotes the expression of SIRT3 by activating mitochondrial complex I in A‐BMMSCs, thereby regulating their mitochondrial energy metabolism and ROS levels. Data are presented as mean ± SD of at least three independent assays for each experiment. Statistically significant differences between groups are set at *p* < 0.05.

After culturing A‐BMMSCs on Y‐ECM or TCPS for 7 days, RNA‐seq analysis was performed to investigate the underlying mechanisms (**Figure** **4**A). The heat map of DEGs after clustering analysis revealed changes in the transcriptome profile following the expansion of A‐BMMSCs on Y‐ECM (Figure 4B). Specifically, a total of 1287 genes were detected, of which 525 genes was significantly down‐regulated and 280 genes were significantly up‐regulated in the Y‐ECM group (Figure 4C). To explore the effects of Y‐ECM on the biological functions of A‐BMMSCs, GO enrichment analysis was performed for DEGs. The top GO terms for biological processes (BP), cellular components (CC), and molecular functions (MF) based on the NES were depicted in Figure 4D, highlighting the significant impact of Y‐ECM on A‐BMMSCs. Several key GO terms were significantly enriched in BP, such as “response to mechanical stimulus”, “cellular response to transforming growth factor beta stimulus”, “extracellular matrix organization”, “mitochondrial respiratory chain complex IV”, “mitochondrial proton‐transporting ATP synthase complex assembly”, “angiogenesis”, and “mitochondrion localization”. In addition, several key GO terms were significantly enriched in CC and MF, such as “collage‐containing extracellular matrix” and “extracellular matrix binding” (Figure 4D). These results indicated that Y‐ECM could regulate multiple functions of A‐BMMSCs, including response to mechanical stimuli, collagen synthesis and secretion, angiogenesis, and mitochondrial function. Subsequently, KEGG enrichment analysis revealed that multiple signaling pathways were potentially activated following Y‐ECM stimulation, encompassing the “PI3K‐Akt signaling pathway”, “focal adhesion”, “cytokine‐cytokine receptor interaction”, “ECM–receptor interaction”, “Wnt signaling pathway”, and “signaling pathways associated with the regulation of stem cell pluripotency” (Figure 4E). Moreover, GSEA was performed to reveal that Y‐ECM activated the “histone deacetylases”, “complex I biogenesis”, “oxidative phosphorylation”, and “antioxidant Activity” in A‐BMMSCs (Figure 4F). The GSEA results revealed that Y‐ECM exerted a positive impact on the energy metabolism and redox homeostasis of A‐BMMSCs.

Considering the intimate association between mitochondrial complex I and NAD^+^ regeneration, alongside the fact that Sirtuins are classified as NAD^+^‐dependent histone deacetylases, we conducted further investigation into the expression of the sirtuin family (SIRT1‐7). Interestingly, RT‐qPCR analysis revealed a significant upregulation of mRNA levels for several members of the SIRT family in A‐BMMSCs in the Y‐ECM group, including *SIRT1* (by 56.5%), *SIRT2* (by 92.6%), *SIRT3* (by 101.1%), *SIRT4* (by 37.5%), and *SIRT7* (by 77.2%) (Figure 4G). Given the growing body of evidence emphasizing the substantial role of SIRT3 in bone formation,^[^ ^18^, ^19^ ^]^ this study was specifically designed to further investigate the regulatory effects of SIRT3 on A‐BMMSCs. Furthermore, we found that compared to the TCPS group, both types of ECM could significantly enhance the expression level of the SIRT3 gene, with the promoting effect of the Y‐ECM group being significantly higher than that of the A‐ECM group (Figure S7A, Supporting Information). Alterations in SIRT3 were verified by changes at the protein level (Figure 4H,I). The above findings suggested that the effect of Y‐ECM on enhancing the mitochondrial energy metabolism and antioxidant activity of A‐BMMSCs may be related to the activation of SIRT3 (Figure 4J).

### 2.5 Silencing of *SIRT3* Abrogated the Protective Effects of Y‐ECM on A‐BMMSCs

**Figure 5:**
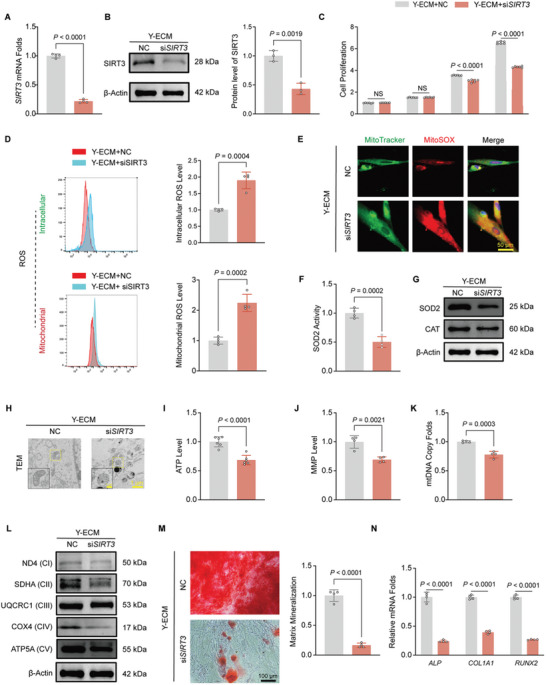
Involvement of SIRT3 in the Y‐ECM‐mediated enhancement of mitochondria functions in A‐BMMSCs. A‐BMMSCs were seeded onto the Y‐ECM‐coated surface and transfected with *SIRT3*‐targeting siRNA (si*SIRT3*) or negative control (NC) siRNA. A) Real‐time PCR confirmed that SIRT3 transcription levels were down‐regulated in si*SIRT3*‐transfected A‐BMMSCs (n = 4 for each group, unpaired two‐tailed Student's *t*‐test). B) Protein levels of SIRT3 in si*SIRT3*‐transfected A‐BMMSCs (n = 3 for each group, unpaired two‐tailed Student's *t*‐test). C) Inhibition of SIRT3 counteracted the effect of Y‐ECM on the proliferation of A‐BMMSCs (n = 6 for each group, unpaired two‐tailed Student's *t*‐test). D) Intracellular and mitochondrial ROS levels in siRNA‐transfected were measured (n = 4 for each group, unpaired two‐tailed Student's *t*‐test). E) Mitochondrial ROS was evaluated using MitoTracker (green) and MitoSOX (red) double‐staining. F) The measurement of SOD2 enzyme activity (n = 4 for each group, unpaired two‐tailed Student's *t*‐test). G) Protein levels of SOD2 and CAT were measured using Western blot (n = 3 for each group, unpaired two‐tailed Student's *t*‐test). H) Silencing SIRT3 abolished the protective effect of Y‐ECM on mitochondrial ultrastructure in A‐BMMSCs. I–K) The ATP levels (n = 6 for each group, unpaired two‐tailed Student's *t*‐test), MMP levels (n = 4 for each group, unpaired two‐tailed Student's *t*‐test) and mtDNA copy number (n = 4 for each group, unpaired two‐tailed Student's *t*‐test) were inhibited in siRNA‐transfected cells. L) Protein levels of ND4, SDHA, UQCRC1, COX4, and ATP5A were measured using Western blot (n = 3 for each group, unpaired two‐tailed Student's *t*‐test). M) Representative images and quantitative analysis of matrix mineralization in si*SIRT3*‐transfected A‐BMMSCs stained by ARS staining (n = 4 for each group, unpaired two‐tailed Student's *t*‐test). N) The effect of SIRT3 inhibition on gene expression of osteoblast‐specific genes (*ALP*, *COL1A1*, and *RUNX2*) on day 21 (n = 4 for each group, unpaired two‐tailed Student's *t*‐test). Data are presented as mean ± SD of at least three independent assays for each experiment. Statistically significant differences between groups are set at *p* < 0.05.

The transfection of *SIRT3* siRNA resulted in a significant reduction of the mRNA and protein levels of SIRT3 by 71.9% (**Figure** **5**A) and 44.9% (Figure 5B), respectively, compared with NC group. The cell proliferation was suppressed in the si*SIRT3* group (Figure 5C). Silencing of *SIRT3* significantly attenuated the effect of Y‐ECM on retarding cellular senescence in A‐BMMSCs (Figure S9, Supporting Information). The antioxidant effect of Y‐ECM was also counteracted, as evidenced by a significant increase in intracellular ROS (89.8% higher than that of the NC group) and mitochondrial ROS (1.2‐fold higher than that of the NC group) (Figure 5D). Immunofluorescence staining further confirmed an increased level of mitochondrial ROS (Figure 5E). Moreover, inhibition of SIRT3 significantly attenuated the activity of SOD2 (Figure 5F) and resulted in a down‐regulation of antioxidant enzymes SOD2 and CAT (Figure 5G; Figure S10, Supporting Information).

The TEM results revealed that the mitochondrial structure of A‐BMMSCs exhibited re‐swelling upon SIRT3 silencing (Figure 5H). Notably, the protective effect of Y‐ECM on mitochondrial energy metabolism in A‐BMMSCs was abolished following *SIRT3* knockdown. Specifically, the si*SIRT3* group exhibited a decrease of 31.5% in ATP production (Figure 5I) and a significant reduction of 30.6% in MMP (Figure 5J) compared to the NC group. Regarding mitochondrial biosynthesis, transfection with *SIRT3* siRNA led to a significant 22.2% decrease in the copy number of mtDNA (Figure 5K). Additionally, the gene expression levels of respiratory chain factors *ND4*, *SDHA*, *UQCRC1*, *COX4* and *ATP5A* were reduced by 29.4%, 75.5%, 44.7%, 40.2%, and 31.4%, respectively, when compared with the NC group (Figure S11A, Supporting Information). The change trend of respiratory factor protein level was consistent with that of mRNA (Figure 5L; Figure S11B, Supporting Information).

To further determine the effect of *SIRT3* knockdown on the multi‐lineage differentiation potentials of A‐BMMSCs, the si*SIRT3*‐transfected cells were cultured in specific differentiation induction medium for 21 days. After an osteogenic induction, the matrix mineralization of the si*SIRT3* group was reduced by 83.5% (Figure 5M). Consistently, compared to the NC group, inhibition of *SIRT3* resulted in a significant reduction of *ALP* mRNA expression by 76.0%, *COL1A1* expression by 60.6%, and *RUNX2* expression by 73.1% (Figure 5N). Subsequently, the level of lipid formation and the transcription level of adipocyte marker genes were significantly reduced in si*SIRT3*‐treated cells (Figure S12A,B, Supporting Information). Moreover, the chondrogenic potential of si*SIRT3*‐transfected cells was significantly impaired, as indicated by the diminished presence of cartilage matrix components and the down‐regulation of chondrocyte marker genes (Figure S12C,D, Supporting Information).

### 2.6 Construction and Characterization of HM@A‐ECM and HM@Y‐ECM

**Figure 6:**
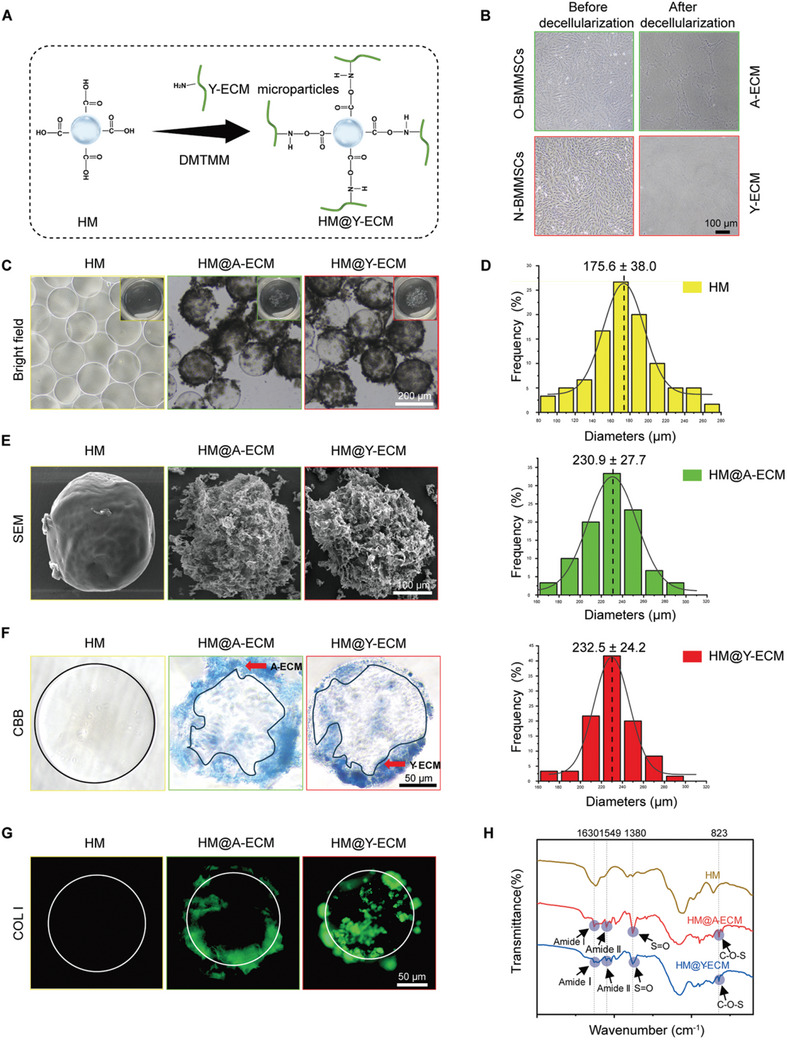
Preparation and characterization of HAMA hydrogel microspheres containing A‐ECM (HM@A‐ECM) or Y‐ECM (HM@Y‐ECM). A) Synthesis of HAMA@ECM used the crosslinking agent DMTMM. B) Representative images of cell monolayers before and after decellularization of BMMSCS from newborn and aged rats. The decellularization process successfully removed the cells to prepare cell‐deposited ECM. C) Optical microscope images of HM, HM@A‐ECM, and HM@Y‐ECM. D) Particle size distribution of HM, HM@A‐ECM, and HM@Y‐ECM. E) Representative SEM images of HM, HM@A‐ECM, and HM@Y‐ECM. F) Representative image of protein distribution on the surface of different HMs when stained with CBB. G) Immunofluorescence staining for COL I. H) FTIR analysis of HM, HM@A‐ECM, and HM@Y‐ECM.

To further evaluate the rejuvenation effect of Y‐ECM on aged bone regeneration in vivo, the Y‐ECM microparticles were conjugated to HAMA HMs using DMTMM as a cross‐linking agent (**Figure** **6**A). First, A‐ECM from aged rats and Y‐ECM from newborn rats were prepared, respectively, and effectively integrated with HMs (Figure 6B,C). The results of particle size analysis confirmed the binding of ECM microparticles to the hydrogel microspheres. The average particle size of the hydrogel microspheres was 175.6 ± 38.0 µm, whereas for HM@A‐ECM and HM@Y‐ECM, it increased to 230.9 ± 27.7 µm and 232.5 ± 24.2 µm, respectively (Figure 6D). SEM observations confirmed the presence of ECM on both HM@A‐ECM and HM@Y‐ECM microspheres (Figure 6E). Notably, pure HMs remained colorless and transparent after Coomassie Brilliant blue (CBB) staining, while a significant amount of blue protein material was observed on the surface of HM@A‐ECM and HM@Y‐ECM (Figure 6F). The presence of COL I was subsequently observed in the HM@A‐ECM and HM@Y‐ECM microspheres through immunostaining (Figure 6G). Finally, the functional groups and molecular conformations of each group were analyzed by FTIR. The results showed that there were absorption peaks of amide I (1630 cm^−1^) and amide II (1549 cm^−1^) in HM@A‐ECM and HM@Y‐ECM microspheres, indicating the presence of collagen proteins (Figure 6H). In addition, two characteristic absorption peaks appeared in the FTIR spectra of HM@A‐ECM and HAMA@Y‐ECM compared with the HM group. One signal at 1380 cm^−1^ was assigned to the stretching vibration of S = O in sulfate, while another signal at 823 cm^−1^ represented the bending vibration of C–O–S in sulfate, which is consistent with the incorporation of a sulfate group.

### 2.7 Effect of HM@Y‐ECM Treatment on Calvarial Defects in Aged Rats

**Figure 7:**
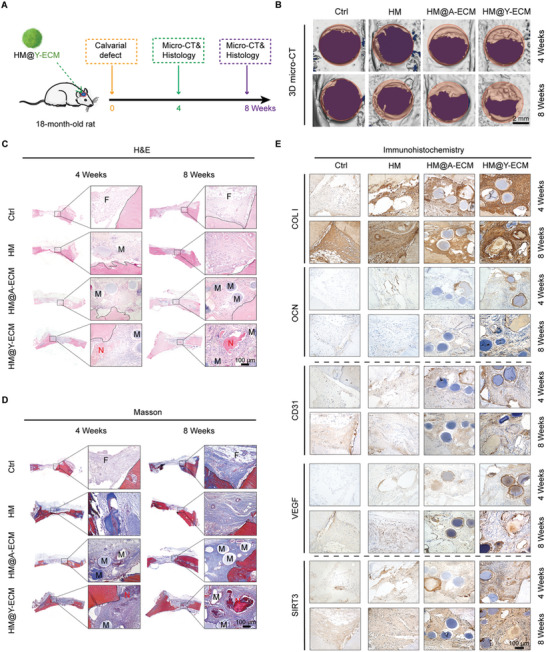
HM@Y‐ECM effectively facilitated vascularized bone regeneration of calvarial defects in aged SD rats. A) Schematic diagram representing the treatment strategy. B) Representative images of the 3D construction from µCT. C,D) Representatives cross sections of the histological evaluation of the defect area at weeks 4 and 8 by H&E and Masson's trichrome staining. (N: new bone tissue; M: microspheres; F: fibrous tissue; Black dotted box: old bone boundary). E) Representative images of immunohistochemistry of COL I, osteocalcin (OCN), CD31, VEGF, and SIRT3 in the newly formed bone tissue. Data are presented as mean ± SD. Statistically significant differences between groups were determined at a threshold of *p* < 0.05.

A calvarial defect model in aged SD rats was established to further evaluate the regenerative effect of YHM@Y‐ECM (**Figure** **7**A). The untreated bone defects were utilized as the control group (CTRL). As shown in Figure 7B, the new bone tissue presented a high‐density shadow in the reconstructed µCT images, and gradually grew from the periphery to the middle of the calvarial defect. There was a progressive increase in new bone formation within the defect area for all groups: CTRL group, HM group, HM@A‐ECM group, and MS@Y‐ECM group (Figure 7B). However, the CTRL group exhibited a slight degree of marginal roughness at both 4 and 8 weeks post‐operatively, indicating suboptimal bone regeneration in the aged rats themselves. The quantitative analysis revealed a significant increase in the percentage of BV/TV in aged rats treated with HM@Y‐ECM, compared to the CTRL group. Specifically, there was a 3.2‐fold and 3.6‐fold increase in the BV/TV at 4 and 8 weeks, respectively (Figure S13A, Supporting Information).

The histological analysis revealed that the CTRL group only exhibited a substantial presence of fibrous tissue bridging the bone margin, further supporting the impaired bone regeneration ability of the aged rats. Compared with the HM group, more new bone tissues were observed in the HM@A‐ECM and HM@Y‐ECM groups. However, the proportion of new bone tissues in the HM@Y‐ECM group was significantly higher than that in the HM@A‐ECM group (Figure 7C,D). The quantification of new bone area at both week 4 and week 8 revealed a consistent trend in bone formation among the groups (Figure S13B, Supporting Information). Additionally, immunohistochemical analysis revealed that in the HM@Y‐ECM group the expression of osteogenic markers (COL I and OCN), angiogenic markers (CD31 and VEGF), and SIRT3 was significantly up‐regulated (Figure 7E; Figure S13C–G, Supporting Information). These results indicate that the HM@Y‐ECM treatment effectively facilitated vascularized bone regeneration at the defect site.

## 3 Discussion

The primary characteristics of aged bone defects encompass a significant decline in the regenerative capacity of bone, accompanied by a reduction in the number and differentiation capacities of BMMSCs present in bone tissue. The impressive performance of mECM in stem cell regeneration, as demonstrated in various cell models, has motivated us to investigate the biological mechanism by which mECM regulates stem cell function.^[^ ^10^, ^25^ ^]^ The present study has identified Y‐ECM as a biomaterial capable of modulating mitochondrial homeostasis of BMMSCs. Specifically, Y‐ECM contains more matrix components that support mitochondrial function compared to A‐ECM and can effectively restore the expression of SIRT3 in A‐BMMSCs. Although ECM has been shown to regulate mitochondrial function, the link between the role of mECM in mitochondrial energy metabolism of BMMSCs and its eventual role in bone formation has remained elusive.^[^ ^26^ ^]^ Here, we identified that mitochondrial energy metabolism regulation, a previously unexplored central link, is the primary mechanism by which Y‐ECM affects the bone formation capacity of A‐BMMSCs and promotes the regeneration of aged bone defects.

Mitochondria, playing an important role in the pathogenesis of age‐related diseases, are increasingly being recognized as a prime target for therapeutic intervention.^[^ ^27^ ^]^ Our results showed that A‐BMMSCs exhibit mitochondrial dysfunction compared to F‐BMMSCs. Some antioxidants, such as vitamin C, are thought to protect mitochondria, but their cellular uptake is limited or they do not accumulate where needed.^[^ ^28^ ^]^ Innovative strategies like mitochondrial transfer, gene editing, and mitochondria‐targeting‐based nanotechnology are also explored to boost the mitochondrial activity.^[^ ^29^ ^]^ However, these therapeutic approaches are restricted by the lack of selectivity in identifying mitochondrial dysfunctional cells, anticipated difficulties of clinical translation, excessive cost of commercialization, and unavoidable safety concerns.^[^ ^30^ ^]^ By comparison, mECM, as a natural material, can not only simulate the natural growth environment of cells but also provide necessary structural support and bioactive signals as well as high biosafety.^[^ ^8^, ^9^, ^31^ ^]^ In this study, we observed a significant improvement in mitochondrial energy metabolism and bone formation potential in A‐BMMSCs cultured on Y‐ECM, demonstrating its potential in the treatment of aged bone defects.

Next, we explored the potential mechanisms through which Y‐ECM regulated the mitochondrial function of BMMSCs. AFM measurement showed that the Y‐ECM had a lower stiffness relative to the A‐ECM. These changes in ECM stiffness are important because they can alter microtubule dynamics, which are critical for mitochondrial function.^[^ ^32^ ^]^ Of note, ECM stiffening has been shown to compromise hair follicle stem cell function during aging, suggesting that Y‐ECM with lower stiffness may aid in protecting mitochondrial function. Additionally, the matrix components of ECM also play an important role in the regulation of A‐BMMSCs. However, the components of cell‐derived ECM are highly intricate and new components are continuously being discovered, such as matrix‐bound nanovesicles (MBVs), which are regarded as a distinct subpopulation of extracellular vehicles (EVs).^[^ ^33^ ^]^ Our proteomic results suggest that Y‐ECM may contain more components that are beneficial for protecting mitochondrial energy metabolism. MBVs may be involved in the protective effects of Y‐ECM on mitochondria, as a recent study has reported that the beneficial effects of young blood were partially mediated by the improvement of mitochondrial energy metabolism through young EVs.^[^ ^34^ ^]^ Future studies are needed to further identify the specific components of Y‐ECM that play a key role in regulating mitochondrial function, with a particular focus on investigating the role of MBVs.

In this study, we found that the gene levels of several SIRT family members in A‐BMMSCs changed significantly during expansion on Y‐ECM, including SIRT1, SIRT2, SIRT3, and SIRT7. Given the pivotal role of SIRT3 in preserving mitochondrial homeostasis and redox balance, we selected SIRT3 as the central subject of our investigation.^[^ ^35^ ^]^ Importantly, the fact that silencing *SIRT3* significantly attenuated the protective effect of Y‐ECM suggests that the catalytic activity of SIRT3 is an important factor in the regeneration capacity of A‐BMMSCs. Notably, our proteomic and transcriptomic data collectively indicate that treatment with Y‐ECM may lead to an elevation in the activity of mitochondrial complex I, which acts as the gatekeeper of the respiratory chain by catalyzing the initial oxidation of NADH. A previous research has confirmed that enhancing the activity of mitochondrial complex I can increase the NAD^+^/NADH ratio, while the deficiency of mitochondrial complex I reduces the ratio, leading to protein hyper‐acetylation.^[^ ^35^, ^36^ ^]^ Interestingly, as an NAD^+^‐dependent deacetylase, SIRT3 can also directly interact with mitochondrial complex I to enhance its activity.^[^ ^37^ ^]^ Therefore, we inferred that the virtuous cycle between SIRT3 and mitochondrial complex I is highly likely to play an important role in the mitochondrial protective effect of Y‐ECM on A‐BMMSCs. However, it should be emphasized that our study does not exclude the potential contributions of other SIRT family members to the Y‐ECM's beneficial effects and our future studies will continue to explore the underlying mechanisms.

In the present study, we designed HM@Y‐ECM to provide growth guidance and osteogenic induction for accelerating aged bone defect repair. As a rejuvenating substrate, the advantage of ECM lies in its capacity to provide an excellent microenvironment for cells, guiding cellular behavior and tissue regeneration.^[^ ^38^ ^]^ More importantly, ECM degradation products have been demonstrated to recruit a substantial number of endogenous progenitor and stem cells to the site of injury, indicating that ECM‐based biomaterials have the potential to promote in situ bone tissue regeneration.^[^ ^39^ ^]^ Unlike tissue‐derived ECM, which presents recognized challenges including pathogen transmission, supply constraints, and donor site morbidity, mECM has more advantages as it can be obtained from autologous sources in a non‐invasive or minimally invasive manner. ^[^ ^10^, ^23^, ^40^ ^]^ In the current study, we ingeniously combined BMMSCs‐derived ECM with biodegradable HAMA photo‐crosslinked hydrogel microspheres to create a novel and advanced bone repair scaffold. This remarkable approach not only incorporates the advantages of hydrogel microspheres (e.g., adjustable mechanical properties and a large specific surface area) into this composite but also offers precise guidance and orientation for endogenous cells to facilitate bone regeneration in vivo. As reported by Deng et al., mECM‐modified poly(lactide‐co‐glycolide) scaffolds not only combate the foreign body response, but also promote bone regeneration by increasing the accumulation of M2 macrophages.^[^ ^41^ ^]^ This is consistent with our results, as evidenced by a significantly greater amount of new and mature bone was formed surrounding the HM@Y‐ECM implant in the calvarial defects. Newly formed blood vessels are critical for bone defect healing by providing essential oxygen and nutrients. Notably, the peri‐implant cells around the HM@Y‐ECM implant had the highest expression of CD31 and VEGF, indicating a large number of new blood vessels. Since mECM is known to promote angiogenesis, the possible synergistic effects of Y‐ECM and HAMA on angiogenesis need to be further investigated.^[^ ^42^ ^]^ Additionally, mECM has demonstrated potential as a promising carrier for bone morphogenetic protein‐2 (BMP‐2), indicating that modification strategies based on mECM may further enhance the bone regeneration capacity of this biomaterial.^[^ ^43^ ^]^

There are several limitations in the current study. One of the challenges is the limited availability of donors for the preparation of mECM. Future research should incorporate a more diverse donor pool, spanning various ages and genders, to enhance the universality and reliability of the study's findings. While mECM, particularly Y‐ECM, has been demonstrated to ameliorate mitochondrial function and facilitate the repair of aged bone defects, the specific matrix components that regulate mitochondrial energy metabolism of A‐BMMSCs need to be fully understood. Notably, our study showed that SIRT3 plays an important role in the Y‐ECM‐mediated regulation of A‐BMMSC multi‐lineage differentiation potentials, yet it lacks the detection of late‐stage differentiation markers. Future studies will compare the in vivo bone formation capabilities of *SIRT3*‐silenced cell groups grown on A‐ECM and Y‐ECM. In addition, we will continue to investigate the molecular mechanisms by which Y‐ECM regulates mitochondrial structure and function through other SIRT family members.

**Figure 8:**
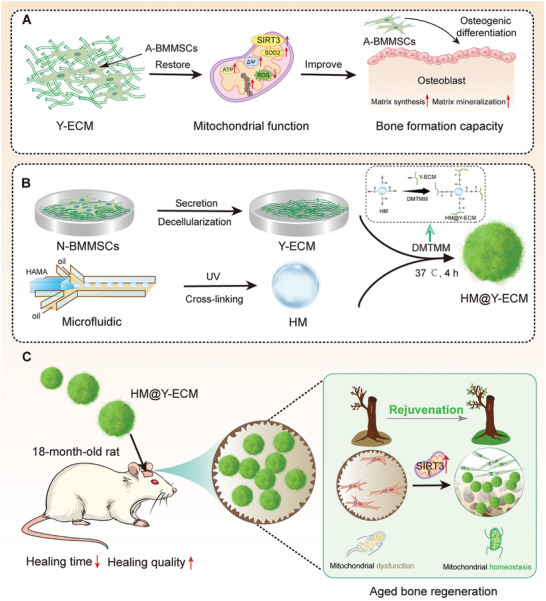
A schematic overview of this study. A) Rejuvenation of A‐BMMSCs by Y‐ECM rescues the impaired mitochondrial functions and restores the redox homeostasis. Consequently, the Y‐ECM‐treated A‐BMMSCs promotes bone formation. B) HM@Y‐ECM composite was constructed through an amidation reaction between HAMA hydrogel microspheres and Y‐ECM under the action of DMTMM. C) HM@Y‐ECM composite was implanted into a calvarial defect of 18‐month‐old rats. HM@Y‐ECM elicits a rejuvenation effect “dead wood coming to life in spring”, significantly enhancing mitochondrial activity within senescent MSCs and effectively promoting the repair of aged bone defects.

In conclusion, we demonstrate that Y‐ECM not only enhanced mitochondrial functions but also improved the multi‐lineage differentiation potentials of A‐BMMSCs. By activating SIRT3, Y‐ECM restored intracellular redox homeostasis and mitochondrial energy metabolism. A novel HM@Y‐ECM composite was developed by integrating Y‐ECM with HAMA microspheres. Upon implantation into a calvarial defect of aged rats, HM@Y‐ECM composite effectively promoted vascularized bone formation (**Figure** **8**). This strategy potentially represents a widely acknowledged cell‐free regenerative approach, offering a promising clinical alternative for the treatment of age‐related bone defects.

## 4 Experimental Section

### Ethical Regulations Statement

All animal experiments were approved by the Ethics Committee of Soochow University, Soochow, China (SUDA20230718A02) and met the requirements of the Guidelines for Care and Use of Laboratory Animals.

### Reagents and Antibodies

Human fetal source BMMSCs (F‐BMMSCs) and A‐BMMSCs were obtained from Cyagen Biosciences (Guangzhou, China). The A‐BMMSCs were obtained from a 44‐year‐old male donor, as confirmed by the supplier's documentation. To ensure a consistent basis for the comparative analysis, both the F‐BMMSCs and A‐BMMSCs were derived from separate single donors. Unless noted otherwise, all chemicals were purchased from Sigma–Aldrich (St Louis, MO, USA).

### Decellularized Extracellular Matrix (DECM) Preparation

A standard procedure for preparing DECM was described previously.^[^ ^8b^ ^]^ The ECM deposited by F‐BMMSCs or neonatal rat BMMSCs (N‐BMMSCs) was termed young ECM (Y‐ECM), while the ECM deposited by A‐BMMSCs or 18‐month‐old Sprague‐Dawley (SD) rat BMMSCs (O‐BMMSCs) was termed aged ECM (A‐ECM). In this study, O‐BMMSCs and N‐BMMSCs were derived from the bone marrow of aged (n = 9) and newborn (n = 9) rats, respectively. Briefly, the tissue culture polystyrene (TCPS) plates were first precoated with 0.2% gelatin for 1 h at 37 °C, followed by sequential treatment with 1% glutaraldehyde and 1 M ethanolamine at room temperature for 30 min each. Subsequently, BMMSCs were seeded onto the pre‐treated cell plates at a density of 5 × 10^3^ cells cm^2^ and cultured in alpha minimum essential medium (α‐MEM) supplemented with 10% fetal bovine serum (FBS), 100 U mL^−1^ penicillin, and 100 µg mL^−1^ of streptomycin (all obtained from Thermo Fisher Scientific, Waltham, MA, USA). Upon reaching 80%–90% confluence, the medium was supplemented with a final concentration of 100 µM L‐ascorbic acid and maintained for an additional 8 days. For decellularization, the cultured cells were subjected to incubation with a solution of phosphate buffered saline (PBS) containing 0.5% Triton X‐100 and 20 mM NH_4_OH (pH = 7.4) at 37 °C for 5 min, followed by treatment with 100 U mL^−1^ DNase I for 1 h at 37 °C. The DECM was subsequently washed three times with PBS, and either utilized immediately in experiments or stored under sterile conditions at 4 °C.

### Characterization of A‐ECM and Y‐ECM

*Immunofluorescence staining*: After fixation with 4% paraformaldehyde, the samples were blocked in 1% bovine serum albumin (BSA) for 1 h, and subsequently incubated overnight with a primary antibody against type I collagen (COL I; 1:200, ABclonal, A1352) at 4 °C. After washing with PBS, the samples were treated with an Alexa Fluor 488 conjugated secondary antibody (1:500, Abcam, ab150077) for 1 h. The fluorescence images were captured using a Zeiss Axiovert 40CFL microscope (Zeiss, Oberkochen, Germany).

*Scanning electron microscope (SEM)*: The samples were immersed in a 2.5% glutaraldehyde solution for 10 min Subsequently, the samples were dehydrated in a series of ethanol solutions with increasing concentrations (50%, 75%, 80%, 95%, and 100%). These samples then underwent critical point drying (CPD300; Leica, Vienna, Austria) and were subsequently subjected to gold sputtering (SC7620; Quorum Technologies, Lewes, UK). The morphology of the samples was photographed using a SEM (S3000N, Hitachi, Tokyo, Japan) with an accelerating voltage of 5 kV.

*Atomic force microscopy (AFM)*: The surface topography and elasticity of the ECMs were analyzed using a nano‐indenter of the AFM (Dimension ICON, Bruker, USA). For the nanomechanics testing, a spherical tip with a radius of 5 nm and a stiffness of 0.4 N m^−1^ (Scanasyst‐air, Nanoworld, Switzerland) was used. Microscopic topography was scanned at a frequency of 1 Hz, while force curves were measured at a frequency of 14 Hz in a contact mode.

*Proteomics analysis*: After collecting by a cell scraper, the DECM were processed into peptides in accordance with a prior research.^[^ ^24^ ^]^ In brief, the DECM samples were initially suspended in an 8 M urea solution (pH = 8) and subjected to reduction using 10 mM dithiothreitol, followed by alkylation and deglycosylation. Subsequently, the samples underwent digestion using Lys‐C in conjunction with mass spectrometry‐grade trypsin. Finally, digestion was quenched by adding trifluoroacetic acid to a final concentration of 0.1%.

Proteomic analysis of ECM polypeptide samples was performed by Luming Biotechnology (Shanghai, China). Briefly, the samples were desalted prior to proteomic analysis and were labeled with tandem mass tag (TMT) (Thermo Fisher Scientific). The separation of peptides was carried out using an 1100 HPLC system (Agilent) equipped with an Agilent Zorbax ExpanD‐C18 column, followed by lyophilization of the separated peptides for mass spectrometry. Subsequently, the Q Exactive mass spectrometer (Thermo) equipped with a Nanospray Flex source (Thermo) was employed to analyze the samples. Full MS scans were obtained within the mass range of 350–1500 m z^−1^, with a mass resolution of 60 000. The sample protein database was meticulously scrutinized for all Q Exactive raw data using Proteome Discoverer (v.2.4). Based on the screening of credible proteins and their data quality control, differential protein analysis was conducted. The criteria for screening differential proteins were a fold change greater than 2 (|logFC| > 1), and the False Discovery Rate (FDR) was used to correct the p‐values, with a corrected P‐value less than 0.05 (FDR < 0.05). The ggplot2 package in R software (version 4.2.2) for Principal Component Analysis (PCA) was used for the creation of volcano plots. By uploading the differential proteins to the Metascape online database with parameters set for Homo species using the Custom Analysis mode, biological enrichment analysis was performed. Additionally, Cytoscape‐v3.9.1 software for visualizing the top 20 enriched pathway cluster modules was used. The ClusterProfiler package was employed for Gene Set Enrichment Analysis (GSEA) on all valid proteins that were not previously filtered, using the reference gene set from the MSigDB database “c5.all.v7.5.1 symbols.gmt”. Each analysis was performed with 1000 permutations, and gene sets with a P‐value less than 0.05, FDR less than 0.25, and a normalized enrichment score (|NES|) greater than 1 were considered significantly enriched. Finally, the ggplot2 package to plot ridge plots to display the GSEA results of each gene set was used.

### In Vitro Culture and Differentiation of BMMSCs

*Cell culture*: BMMSCs were plated at 3000 cells cm^2^ on three different substrates: TCPS, A‐ECM, Y‐ECM in growth medium (α‐MEM supplemented with 10% FBS, 100 U mL^−1^ of penicillin, and 100 µg mL^−1^ of streptomycin) and maintained in a humidified incubator with 5% CO_2_ at 37 °C. The medium was changed every 3 days, and the cellular morphology was observed using an Olympus I X 51 microscope (Olympus Corporation, Tokyo, Japan).

*Cell proliferation analysis*: Cells were cultured onto either TCPS or DECM at a density of 1000 cells cm^2^. On days 1, 3, 5, and 7, the cells were incubated with a 10% Cell Counting Kit‐8 (CCK‐8) solution (Beyotime Institute of Biotechnology, Haimen, China) at 37 °C for 1 h. The absorbance at a wavelength of 450 nm was quantified using a microplate spectrophotometer (BioTek, Winooski, VT, USA).

*Senescence‐associated β‐galactosidase (SA‐β‐gal) assay*: SA‐β‐gal staining was performed using a commercially available kit (Yudo Institute of Biotechnology, China) according to the manufacturer's instructions. Briefly, cells were washed with PBS and fixed for 5 min at room temperature. The cells were then incubated in an SA‐β‐gal detection solution overnight in a dark 37 °C incubator without CO_2_. To determine the percentage of SA‐β‐gal‐positive cells, digital images of 10 randomly selected fields were captured using an Olympus IX51 microscope, encompassing a minimum of 200 cells.

### Cell Differentiation Assays

*Osteogenic differentiation*: For osteogenic differentiation, cells were cultured in an osteogenic differentiation medium (DMEM containing 10% FBS, 100 U mL^−1^ penicillin, 100 µg mL^−1^ streptomycin, 0.2 mM L‐ascorbic acid, 100 nM dexamethasone, and 10 mM β‐glycerophosphate) for 21 days. The production of calcium deposits was stained by Alizarin red S (ARS) solution. Following the fixation with 4% paraformaldehyde, 0.1% ARS working solution (G1452, Solarbio) was added into each well and incubated for 15 min. Subsequently, calcium salt deposition was observed using an Olympus IX51 microscope. The calcium‐chelated stain was extracted by incubation with 5% perchloric acid, followed by measurement at 420 nm using a PowerWave XS spectrophotometer (BioTek).

*Adipogenic differentiation*: For adipogenic differentiation, cells were cultured in an adipogenic differentiation medium (DMEM containing 10% FBS, 100 U mL^−1^ penicillin, 100 µg mL^−1^ streptomycin, 1 µM IBMX, 1 µM dexamethasone, 100 µM indomethacin, and 10 µg mL^−1^ insulin) for 21 days. The formation of oil droplets within the cells was stained by Oil red O. Following the fixation with 4% paraformaldehyde, 0.3% Oil red O working solution was added into each well and incubated for 30 min. Subsequently, stained droplets in the adipocytes were visualized using an Olympus IX51 microscope. For quantification, absorbance at 510 nm was measured after dissolving the stained cells with 100% isopropanol for 30 min.

*Chondrogenic differentiation*: For chondrogenic differentiation, cells were cultured in a chondrogenic differentiation medium (DMEM‐high glucose containing 2% FBS, 100 U mL^−1^ penicillin, 100 µg mL^−1^ streptomycin, 1 × Insulin‐Transferrin‐Selenium‐Solution, 100 µM L‐ascorbic acid, 40 µg mL^−1^ proline, 100 nM dexamethasone) supplemented with 10 ng mL^−1^ transforming growth factor beta‐1 (TGFβ1, PeproTech Inc., Rocky Hill, NJ, USA) for 21 days. The production of sulfated glycosaminoglycans (GAGs) was stained by Alcian blue. Following the fixation with 10% neutral buffered formalin, Alcian blue working solution (G2542, Solarbio) was added into each well and incubated for 30 min. Subsequently, stained GAGs were observed using an Olympus IX51 microscope. Alcian blue staining was quantified by extracting the color in 100% dimethyl sulfoxide (DMSO, Sigma–Aldrich) and measuring the absorbance at 610 nm.

### *SIRT3* Small Interfering RNA (siRNA) Interference

To investigate the role of SIRT3 in regulating mitochondrial functions, cells in 12‐well plates were transfected with *SIRT3*‐targeting siRNA (GenePharma, Shanghai, China) or with negative control siRNA (NC) at a final concentration of 100 nM using Lipofectamine 3000 (Thermo Fisher Scientific). The targeting sequence for *SIRT3* siRNA was as follows: 5′‐GCUUGAUGGACCAGACAAATT‐3′ (sense) and 5′‐UUUGUCUGGUCCAUCAAGCTT‐3′ (antisense). After 6 h, the culture medium was replaced with a fresh growth medium.

### Assessment of Mitochondrial Function and Antioxidant Capacity

*Mitochondrial membrane potential (MMP)*: For the assessment of MMP level (ΔΨm), cells were incubated with JC‐1 solution (C2006, Beyotime) at 37 °C in 5% CO_2_ for 20 min. After washing with PBS, the Guava easyCyte Flow Cytometer (Millipore, Boston, MA, USA) was utilized to detect aggregates in polarized mitochondria (red) and monomers in depolarized mitochondria (green), respectively. The MMP levels were calculated by determining the ratio of red to green fluorescence intensity.

*ATP concentration detection*: Cells cultured on different substrates were completely lysed. The ATP content was measured using an Enhanced ATP Assay Kit (S0027, Beyotime), following the manufacturer's guidelines. The supernatant was mixed with the ATP detection working solution in a black 96‐well plate for luminescence analysis utilizing a Centro LB 960 (Berthold Technologies, Germany). To normalize the ATP levels, the protein concentration of each sample was determined using a BCA protein concentration assay kit (Beyotime).

*Transmission electron microscope (TEM)*: Samples were fixed in 2.5% glutaraldehyde overnight at 4 °C and incubated in 1% osmic acid solution on ice for 1 h. After a series of ethanol washes, the samples were embedded with Araldite (TedPella, Redding, CA, USA). Each sample was sectioned using Leica Ultracut and stained with solution of 2% lead citrate and 2% uranyl acetate at 37 °C in the dark. Images were captured using a TEM (TECNAI G2 F20, FEI, Hillsboro, Oregon, USA) to observe mitochondrial structure.

*Assessment of intracellular and mitochondrial ROS levels*: For intracellular ROS, cells were incubated in 10 µM 2′,7′‐dichlorofluorescein diacetate solution (DCFH‐DA, S0033, Beyotime) at 37 °C for 20 min. For mitochondrial ROS, cells were incubated in 3 µM MitoSOX Red solution (Thermo Fisher Scientific) at 37 °C for 20 min. Subsequently, the samples were analyzed using a Guava EasyCyte Flow Cytometer and the FlowJo 10.8.1 software (TreeStar, San Carlos, CA, USA). The mitochondrial ROS was further visualized by colocalizing MitoSOX Red and Mitotracker Green (C1048, Beyotime) intensity, which was detected using a Zeiss Axiovert 40CFL microscope.

*Superoxide dismutase 2 (SOD2) activity assay*: The activity of SOD2 was analyzed using a commercially available kit (S0103, Beyotime), in accordance with the manufacturer's instructions. The total protein concentration was determined using a BCA protein Assay kit (Beyotime). Subsequently, samples were incubated with an SOD1 inhibitor and mixed with WST‐8 working solution, followed by a 30 min incubation at 37 °C. The absorbance was measured at 450 nm using a microplate reader (BioTek), and the activity of SOD2 was normalized to the total protein levels.

*Quantitative reverse transcription PCR (RT‐qPCR) and mitochondrial DNA (mtDNA)*: Total RNA was extracted using the TRIzol reagent (Thermo Fisher Scientific), followed by the synthesis of complementary DNA (cDNA) from 1 µg of the purified RNA using the RevertAid First Strand cDNA Synthesis Kit (Thermo Fisher Scientific). RT‐qPCR was then performed with the iTap Universal SYBR Green Supermix kit (Bio‐Rad, Hercules, CA, USA) on a CFX96TM RT‐qPCR System (Bio‐Rad). The relative expressions of target genes were determined using the 2^−ΔΔCT^ method with glyceraldehyde‐3‐phosphate dehydrogenase (*GAPDH*) as the housekeeping gene. Total DNA was extracted using a genomic DNA extraction kit (Vazyme, China). The relative content of mtDNA was calculated by normalizing mitochondrial ND1 gene (mtND1) levels to β‐globin levels using RT‐qPCR. The primer sequences used in this study were listed in Table S1 (Supporting Information).

### Western Blot Analysis

Total protein was extracted using ice‐cold radioimmunoprecipitation assay (RIPA) buffer containing protease inhibitor cocktails, and the protein concentration was determined using a BCA Protein assay kit (Beyotime). Equivalent amounts of denatured protein lysate were separated by electrophoresis in a 10% sodium dodecyl sulfate‐polyacrylamide gel (SDS‐PAGE), and subsequently transferred to a nitrocellulose membrane (Beyotime). The membranes were blocked at 37 °C for 30 min with blocking buffer (Beyotime) and incubated overnight at 4 °C with properly diluted primary antibodies. The information on primary antibodies was provided in Table S2 (Supporting Information). The membranes were further incubated with horseradish peroxidase–conjugated secondary antibodies at 37 °C for 1 h and visualized with an enhanced chemiluminescence solution (Vazyme). All bands on the membranes were detected using a chemical luminescence imaging system (Clinx Science Instruments Co., Ltd, Shanghai, China), and the gray values of the bands were quantitatively assessed using ImageJ software (National Institutes of Health, Bethesda, MD, USA).

### RNA Sequencing

A‐BMMSCs were cultured on TCPS and Y‐ECM for 7 days, respectively. Total RNA was isolated and extracted using the TRIzol reagent (Thermo Fisher Scientific). The RNA purification, library construction, and sequencing were all completed by OE Biotech Co., Ltd. (Shanghai, China). The integrity was assessed using the Agilent 2100 Bioanalyzer (Agilent Technologies, Santa Clara, CA, USA). Transcriptome libraries were constructed using the VAHTS Universal V6 RNA‐seq Library Preparation Kit (Illumina, San Diego, CA, USA) according to the manufacturer's instructions. Subsequently, these libraries were sequenced on an Illumina Novaseq 6000 platform. After obtaining raw reads in fastq format, low‐quality reads were processed and removed using the fastp software to obtain clean reads for subsequent data analysis. Reference genome alignment was performed with HISAT2 software, and gene expression levels (FPKM) were calculated, and read count information for each gene was obtained using HTSeq‐count. Differential expression analysis of the transcriptome data was conducted using the Limma package in R software (version 4.2.2), with the criteria for screening differential genes being a fold change greater than 2 (|logFC| > 1), and the P‐values were corrected using FDR, ensuring the corrected P‐value was less than 0.05 (FDR < 0.05). Volcano plots of differentially expressed data were drawn using the ggplot2 package, and heat maps of the gene matrix were created with the ComplexHeatmap package. Gene Ontology (GO) enrichment analysis and Kyoto Encyclopedia of Genes and Genomes (KEGG) enrichment analysis were performed using the ClusterProfiler software package. GO functional annotation mainly includes three aspects: molecular function (MF), cellular component (CC), and biological process (BP) that genes were involved in. GO and KEGG pathways with significant enrichment were selected using Fisher's exact test with a P‐value less than 0.05. Enrichment analysis results were visualized using the ggplot2 package. Gene set enrichment analysis (GSEA) was conducted on all unfiltered differential genes using the ClusterProfiler package, with data sourced from the MSigDB database. Gene sets with a P‐value less than 0.05, FDR less than 0.25, and a normalized enrichment score (|NES|) greater than 1 were considered significantly enriched.

### HAMA Synthesis

500 mg of HA (MW = 100 kDa, Xianjue New Material Technology Co. Ltd., Suzhou, China) was dissolved in 25 mL of deionized water with a magnetic stir bar for 20 min at room temperature to ensure complete dissolution. Subsequently, a microfluidic pump was used to slowly add dropwise 5 M NaOH to the HA solution until the pH reached 8.5. Methacrylic anhydride (MA, Aladdin, Shanghai, China) was then added dropwise into the HA solution with 5‐fold excess of the amount of HA. The reaction was allowed to carry out on ice for 4 h while maintaining the pH between 7.5–8.5 by adding additional drops of 5 M NaOH as necessary. Next, the solution underwent dialysis against deionized water for 4 days and subsequently freeze‐dried to obtain solid HAMA product. Finally, the purity of the lyophilizate was assessed using proton nuclear magnetic resonance spectroscopy (^1^H NMR, 600 MHz, Bruker, Germany).

### Fabrication and Characterization of HAMA HMs

The HAMA microspheres were fabricated using a microfluidic device, in which an aqueous phase containing 5% (w/v) HAMA solution and 0.5% (w/v) LAP was emitted along with an oil phase consisting of 5% (w/w) Span80 in paraffin oil. Subsequently, solid HAMA microspheres were formed by cross‐linking under ultraviolet radiation for 30 min. Then, the microspheres were sequentially washed with 75% ethanol and isopropanol, followed by storage in deionized water prior to use. The morphology and size of the purified HAMA microspheres were observed using an inverted phase‐contrast microscope, while the microstructure of lyophilized samples was examined using a SEM (S3000N, Hitachi, Tokyo, Japan). Additionally, chemical bond formation in the prepared microspheres was analyzed using Fourier‐transform infrared spectroscopy (FTIR; Bruker, Horiba, Germany). The microspheres were measured within a wavelength range of 400 to 4000 cm^−1^ with a resolution of 0.4 cm^−1^, and each measurement was scanned 128 times.

### Preparation of Arg‐Gly‐Asp (RGD) Peptide‐Modified HAMA Microspheres

To confer cell adhesion properties to HAMA microspheres, the RGD peptide (Sener Bio, Hefei) was introduced onto hydrogel microspheres. Briefly, 10 mg of 4‐(4,6‐Dimethoxy‐1,3,5‐triazin‐2‐yl)‐4‐methylmorpholinium chloride (DMTMM,) and RGD peptides were dissolved in 1 mL of deionized water by magnetic stirring, respectively. Then, the HAMA microspheres were initially soaked in 10 mg mL^−1^ DMTMM solution with gentle stirring for 45 min. Subsequently, the microspheres were gathered via centrifugation at 1200 rpm for 5 min and rinsed with deionized water. The microspheres were then immersed in the RGD solution (10 mg mL^−1^) for 4 h with gentle stirring. Finally, the RGD‐conjugated HAMA microspheres were rinsed with deionized water and stored in deionized water prior to utilization.

### Fabrication and Characterization of HM@ECM

DECM was carefully collected from 55 cm^2^ culture dishes using a cell scraper. Subsequently, the DECM was frozen overnight at −80 °C and then subjected to freeze‐drying until the polymer was completely dry (a process lasting 3–4 days). In order to obtain ECM particles, the resulting lyophilized ECM was thoroughly pulverized using a grinding rod. Subsequently, 10 mg of the pulverized powder was suspended in 1 mL of deionized water and vigorously agitated. Simultaneously, the HAMA HMs were immersed in deionized water containing 1% (w/v) DMTMM for 45 min and rinsed with deionized water. Next, the HMs were mixed with the ECM suspension on a shaker for 4 h. The presence of the ECM on the HMs was observed using an inverted phase‐contrast microscope, while changes in particle size were quantified utilizing ImageJ software. To enable direct observation, the morphology of ECM‐grafted HMs was examined using an SEM equipment. To further demonstrate the grafting of ECM proteins onto the HMs, the distribution of ECM proteins on the HMs was visualized by Coomassie brilliant blue (CBB) staining. In addition, COL I was identified by immunofluorescence staining. Furthermore, FTIR analysis (Bruker, Horiba, Germany) was conducted to provide evidence for chemical bond formation between ECM and HMs.

### Animal Experiments

*Ectopic bone formation assay*: The bone formation capacity of DECM‐pretreated A‐BMMSCs was evaluated using a murine dorsal subcutaneous pocket model. A total of 12 male BALB/c nude mice, aged 6 weeks, were purchased from Beijing Vital River Laboratory Animal Technology Co., Ltd (Beijing, China). A‐BMMSCs were cultured on three different substrates, namely TCPS, A‐ECM, and Y‐ECM, followed by in vitro osteogenic induction for 7 days. The collected cells, numbering 1 × 10^6^, were then seeded onto RGD‐modified HAMA microspheres and implanted subcutaneously into the back of the nude mice. After an 8‐week period of implantation, all subcutaneous implants were isolated and fixed overnight in a 4% paraformaldehyde solution. Subsequently, histological and immunohistochemical analyses were conducted in accordance with established protocols.

*Rat calvarial defect model constructing and implantation*: A total of 32 male SD rats, aged 18 months, were administered anesthesia through an intraperitoneal injection of pentobarbital (3.5 mg/100 g). Subsequently, the rats' skulls were thoroughly shaved and disinfected, and a longitudinal incision of ≈2.5 cm in length was made at the center of the surgical site. The soft tissue was then meticulously separated until the skull was exposed. Two bilateral defects were surgically created using a dental trephine measuring 5 mm in diameter. Subsequently, each defect was thoroughly rinsed and hemostasis was achieved. Following this, hydrogel microspheres were implanted to effectively cover the defect area. The control group did not receive any material. Following the suturing of incisions, all animals were placed on warm sheets to facilitate recovery and subsequently transferred to the animal feeding room for postoperative care. In order to mitigate the risk of infection, all animals were administered intramuscular injections of penicillin solution for 3 days.

### Micro‐Computed Tomography (µCT) Analysis

At 4 and 8 weeks post‐surgery, all animals were humanely euthanized, and the calvarial tissues were collected for subsequent analysis. The collected specimens were then fixed in a 4% formaldehyde solution for 48 h at room temperature. Subsequently, the extracted calvarial bone tissues were subjected to scanning using a µCT machine (SkyScan, SkyScan 1176, Belgium) with parameters set at 65 kV, 385 mA, and a 1 mm Al filter. The CTan software (Skyscan) was employed to obtain bone volume/total volume (BV/TV) data, and the scanned cranial samples were further subjected to 3D reconstruction using Mimic software.

### Histological and Immunohistochemical Analysis

For the purpose of histological and immunochemical analysis, the subcutaneous and in situ implanted samples were collected and subsequently fixed in a 4% paraformaldehyde solution. The subcutaneous samples were fixed for one day, while the in situ samples were fixed for two days. Furthermore, the calvarial tissues were fixed and then subjected to decalcification by gently shaking them in a 10% Ethylene Diamine Tetraacetic Acid (EDTA) solution for 4 weeks. Following decalcification, the specimens were dehydrated using a gradient ethanol method and subsequently embedded in paraffin. The resulting samples were then sliced into 7 µm (for the subcutaneous specimens) and 5 µm (for the in situ specimens) sections using a slicer manufactured by Leica. To evaluate bone regeneration, the staining of each sample was conducted using hematoxylin and eosin (H&E) and Masson's trichrome staining (Solaibao, China) following the manufacturer's protocol. The stained samples were observed under a Zeiss microscope (Zeiss Axiovert 40CFL, Oberkochen, Germany) and images were captured using bright field illumination. For immunohistochemical staining, the sections were subjected to antigen retrieval and subsequently incubated with primary antibodies overnight at 4 °C. The primary antibody information was presented in Table S2 (Supporting Information). The immunostaining samples were visualized with the HRP/DAB kit (CK0062, Signal way Antibody, College Park, MD, USA) on the second day. The quantification of the immunohistochemical staining results was performed using ImageJ software.

### Statistical Analysis

Statistical analysis was conducted using GraphPad Prism 9.0 Software (GraphPad Software, San Diego, CA, USA). *P* values were determined using the two‐tailed Student's *t*‐test for comparisons between two groups or one‐way analysis of variance (ANOVA) with a Tukey's post hoc test for comparisons among three or more groups. A significance level of *p* < 0.05 was considered statistically significant. All quantitative data were presented as mean ± standard deviation (SD).

## Conflict of Interest

The authors declare no conflict of interest.

## Supporting information



Supporting Information

## Data Availability

The data that support the findings of this study are available in the supplementary material of this article.
