# Supplementary material for: Youthful Stem Cell Microenvironments: Rejuvenating Aged Bone Repair Through Mitochondrial Homeostasis Remodeling
Source: Adv Sci (Weinh). 2025 Jan 17;12(10):2409644. doi: 10.1002/advs.202409644 (PMC11905074; doi:10.1002/advs.202409644)
Supplement: Supplementary file 1 — Supporting Information [file ADVS-12-2409644-s001.docx]

Supporting Information for

**Youthful Stem Cell Microenvironments: Rejuvenating Aged Bone Repair through Mitochondrial Homeostasis Remodeling**

Xinfeng Zhou ^a,1^, Xin Tian ^a,1^, Jianan Chen ^a^, Yantong Li ^a^, Nanning Lv ^a^, Hao Liu ^a^, Tao Liu ^a^, Huilin Yang ^a^, Xi Chen ^c,^*, Yong Xu ^a,b,^*, Fan He ^a,^*

^a^ Department of Orthopaedics, The First Affiliated Hospital of Soochow University, Orthopedic Institute, MOE Key Laboratory of Geriatric Diseases and Immunology, Suzhou Medical College, Soochow University, Suzhou, 215000, Jiangsu, China

^b^ Department of Orthopaedics, The Third Affiliated Hospital of Soochow University, Changzhou, 213000, Jiangsu, China

^c^ Department of Pathology, The Third Affiliated Hospital of Soochow University, Changzhou, 213000, Jiangsu, China

^1^ Xinfeng Zhou and Xin Tian contributed equally to this work.

**Corresponding Authors:**

*Xi Chen, Department of Pathology, The Third Affiliated Hospital of Soochow University, No.185 Juqian Rd., Changzhou, 213000, Jiangsu, China. Email: chenxi1124@suda.edu.cn

*Yong Xu, Ph.D., Orthopaedic Institute, Medical College, Soochow University, No. 178 East Ganjiang Road, Suzhou 215000, Jiangsu, China. Telephone: +86-519-68870891; Fax: +86-519-86621235; Email: yxu1615@suda.edu.cn

*Fan He, Ph.D., Orthopaedic Institute, Medical College, Soochow University, No. 178 East Ganjiang Road, Suzhou 215000, Jiangsu, China. Telephone: +86-512-67781386; Fax: +86-512-67781386; Email: [fanhe@suda.edu.cn](mailto:fanhe@suda.edu.cn)


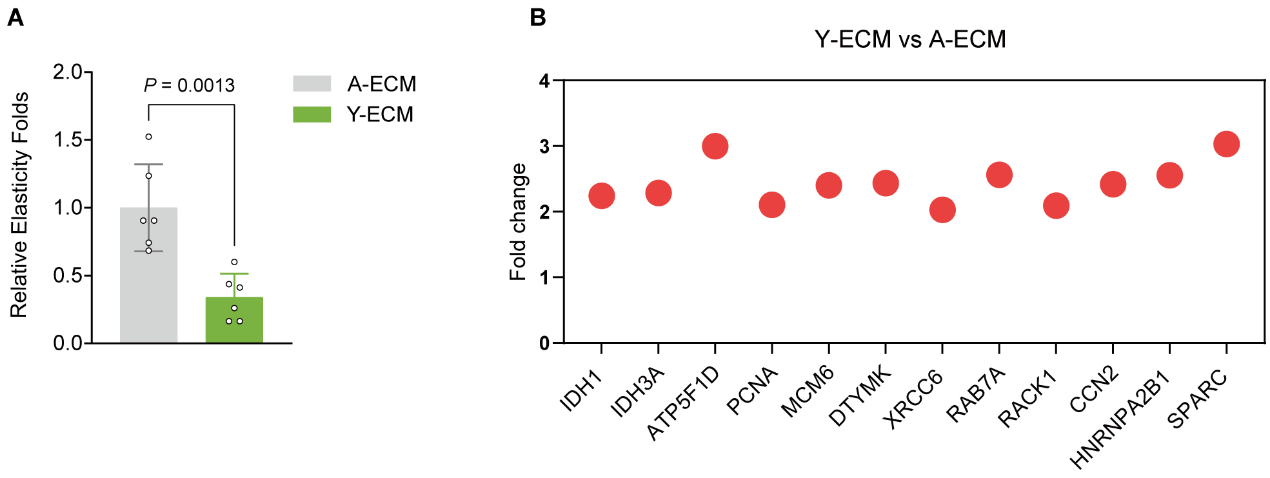


**Fig. S1. Comparison of matrix stiffness and composition between Y-ECM and A-ECM**. A) The elasticity of A-ECM and Y-ECM was measured by AFM (n = 6 for each group, unpaired two-tailed Student’s *t*-test). B) The protein levels of ECM components associated with mitochondrial homeostasis and cellular senescence. Data are presented as mean ± SD of six independent experiments for elasticity assays. Statistically significant differences between groups were determined at a threshold of *P* < 0.05.


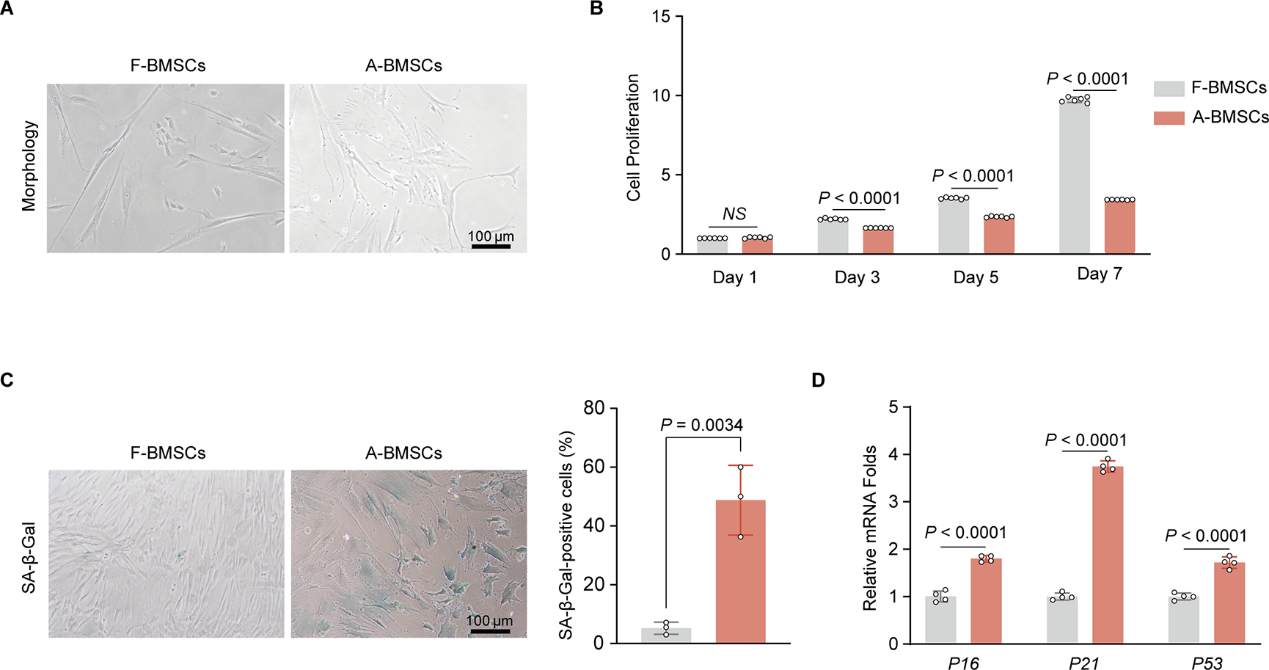


**Fig. S2. Comparison of self-renewal abilities between F-BMMSCs and A-BMMSCs.** A) Compared with A-BMMSCs, F-BMMSCs exhibited smaller cell morphology. B) Cell proliferation of A-BMMSCs and F-BMMSCs (n = 6 for each group, unpaired two-tailed Student’s t-test). C) Representative SA-β-Gal staining images and quantification of F-BMMSCs and A-BMMSCs (n = 3 for each group, unpaired two-tailed Student’s t-test). D) The mRNA expression levels of senescence-related genes (*P16*, *P21*, *P53*) were quantified using qRT-PCR (n = 4 for each group, unpaired two-tailed Student’s t-test). Data are presented as mean ± SD of at least three independent assays for each experiment. Statistically significant differences between groups are set at *P* <0.05.


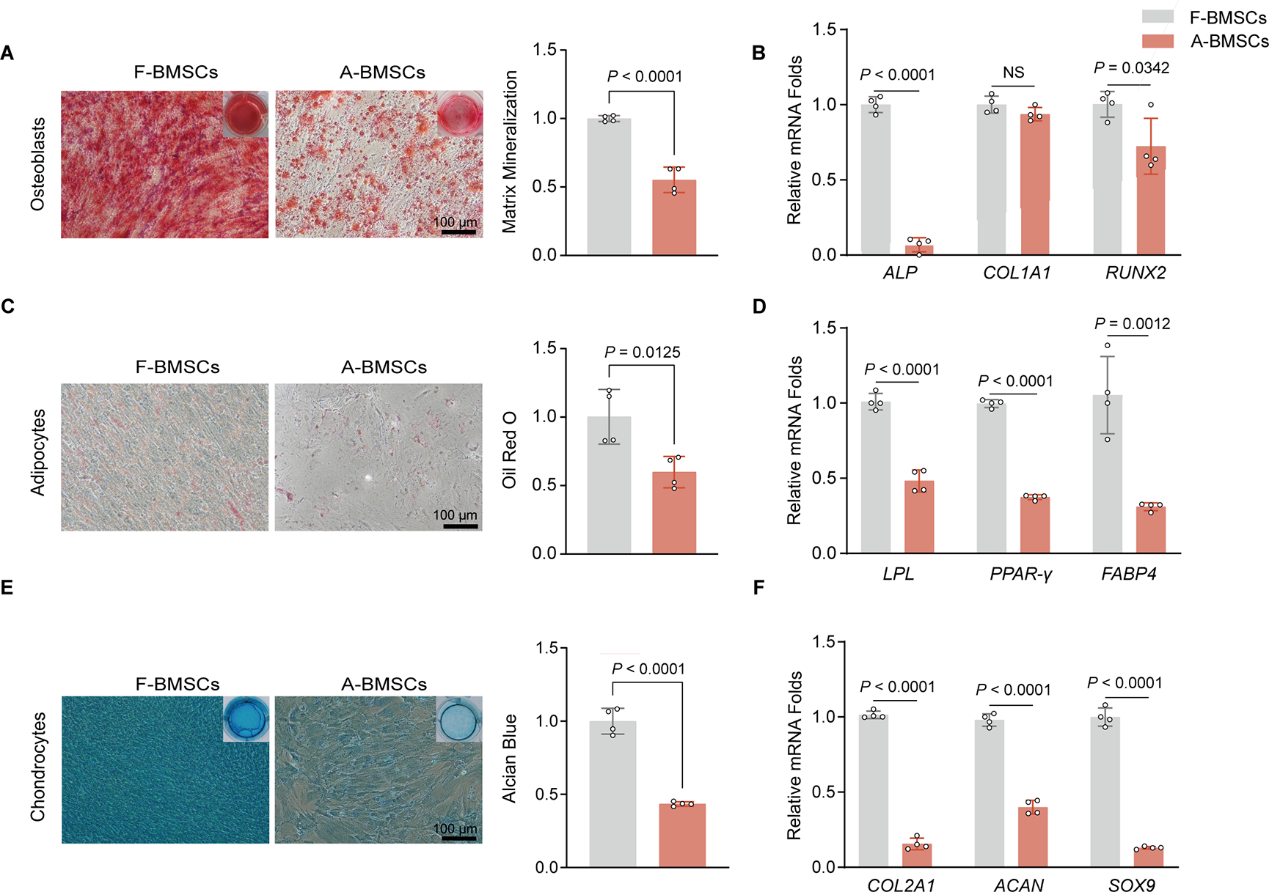


**Fig. S3. Comparison of multi-lineage differentiation potentials of F-BMMSCs and A-BMMSCs.** A) Representative images and quantitative analysis of ARS staining to evaluate matrix mineralization (n = 4 for each group, unpaired two-tailed Student’s *t*-test). B) Osteoblast-specific gene expression: *ALP*, *COL1A1*, and *RUNX2* (n = 4 for each group, unpaired two-tailed Student’s *t*-test). C) Representative images and quantitative analysis of Oil red O staining to examine lipid formation in mature adipocytes (n = 4 for each group, unpaired two-tailed Student’s *t*-test). D) Adipocyte-specific gene expression: *LPL*, *PPAR-γ*, and *FABP4* (n = 4 for each group, unpaired two-tailed Student’s *t*-test). E) Representative images and quantitative analysis of Alcian blue staining to evaluate the content of sulfated GAGs (n = 4 for each group, unpaired two-tailed Student’s *t*-test). F) Chondrocyte-specific gene expression: *COL2A1*, *ACAN*, and *SOX9* (n = 4 for each group, unpaired two-tailed Student’s *t*-test). Data are presented as mean ± SD of four independent assays for each experiment. Statistically significant differences between groups are presented by *P* < .05.

**
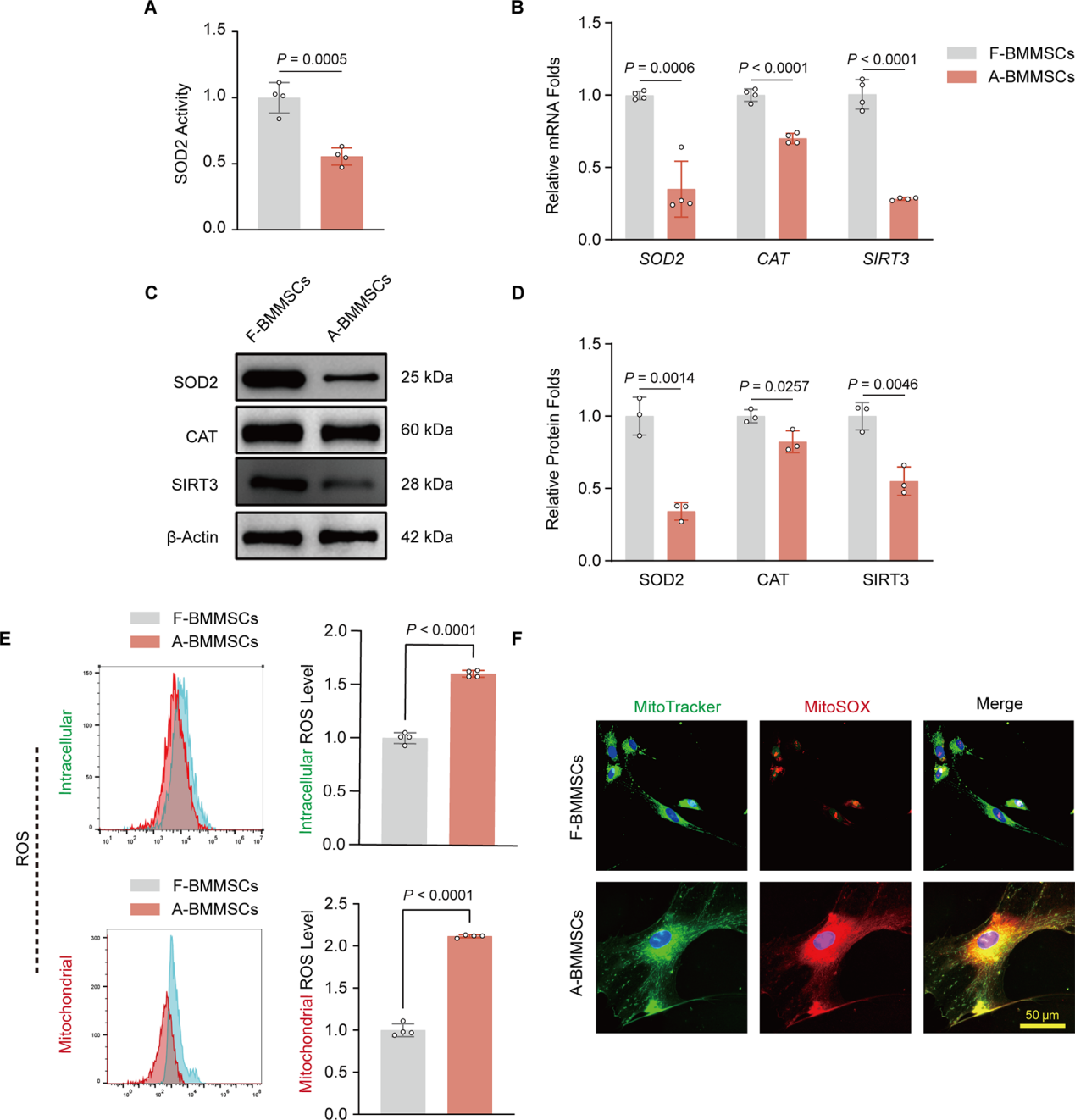
**

**Fig. S4. Comparison of intracellular antioxidant functions of F-BMMSCs and A-BMMSCs.** A) SOD2 activity of F-BMMSCs and A-BMMSCs (n = 4 for each group, unpaired two-tailed Student’s *t*-test). B) Antioxidant gene expression: *SOD2*, *CAT*, and *SIRT3* (n = 4 for each group, unpaired two-tailed Student’s *t*-test). C, D) Quantification of SOD2, CAT, and SIRT3 preoteins in F-BMMSCs and A-BMMSCs (n = 3 for each group, unpaired two-tailed Student’s *t*-test). E) Intracellular and mitochondrial ROS levels (n = 4 for each group, unpaired two-tailed Student’s *t*-test). F) Representative immunofluorescence images by MitoTracker (green) and MitoSOX (red) double-staining. Cell nuclei were stained by DAPI. Data are presented as mean ± SD of at least three independent assays for each experiment. Statistically significant differences between groups are set at *P* <0.05.


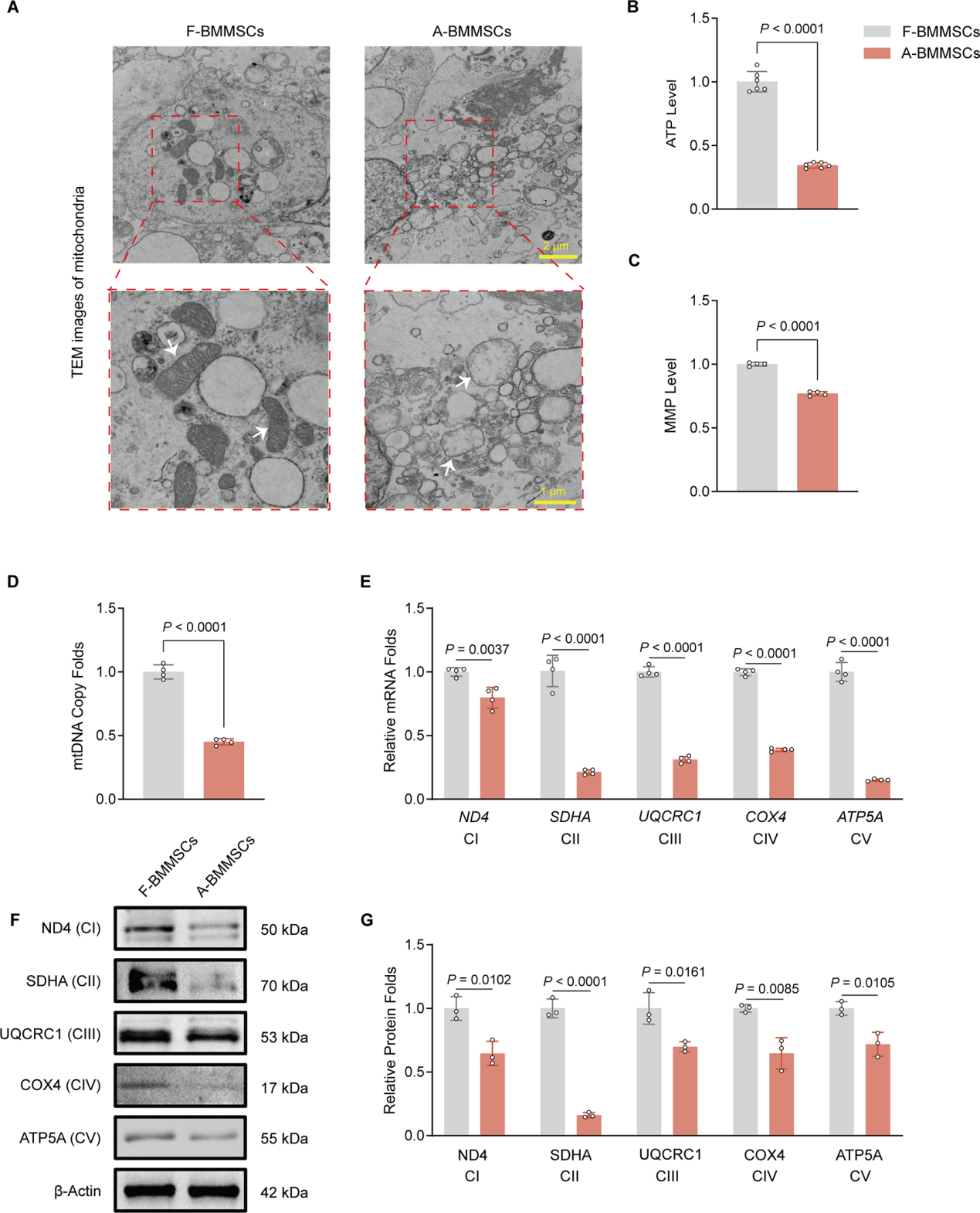


**Fig. S5. Comparison of mitochondrial functions between F-BMMSCs and A-BMMSCs.** A) Representative TEM images of mitochondrial ultrastructure. (white arrow: mitochondria). B-D) The ATP levels (n = 6 for each group, unpaired two-tailed Student’s *t*-test), MMP levels (n = 4 for each group, unpaired two-tailed Student’s *t*-test), and mtDNA copy number (n = 4 for each group, unpaired two-tailed Student’s *t*-test) of F-BMMSCs and A-BMMSCs. E) The mRNA expression of mitochondrial respiratory chains factors including *ND4*, *SDHA*, *UQCRC1*, *COX4*, and *ATP5A* in F-BMMSCs and A-BMMSCs (n = 4 for each group, unpaired two-tailed Student’s *t*-test). F, G) Protein levels of ND4, SDHA, UQCRC1, COX4, and ATP5A in F-BMMSCs and A-BMMSCs (n = 3 for each group, unpaired two-tailed Student’s *t*-test). Data are presented as mean ± SD of at least three independent assays for each experiment. Statistically significant differences between groups are set at *P* < 0.05.


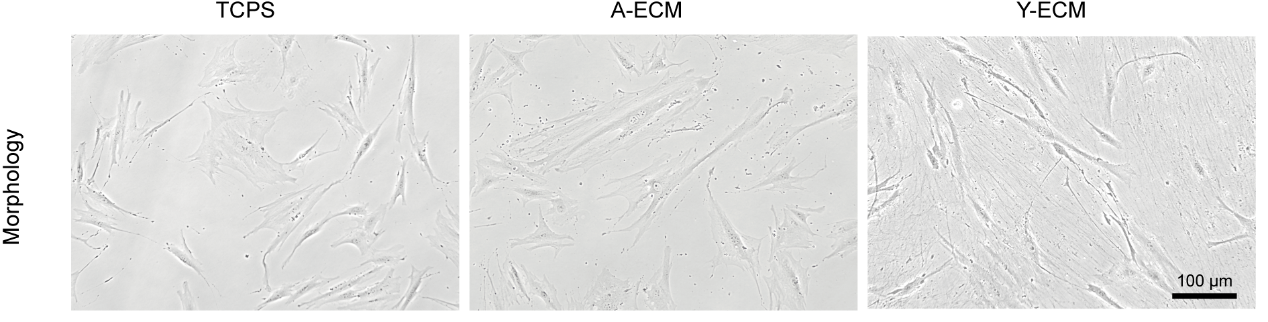


**Fig. S6. The effects of TCPS, A-ECM and Y-ECM on cell morphologies of expanded A-BMMSCs**.


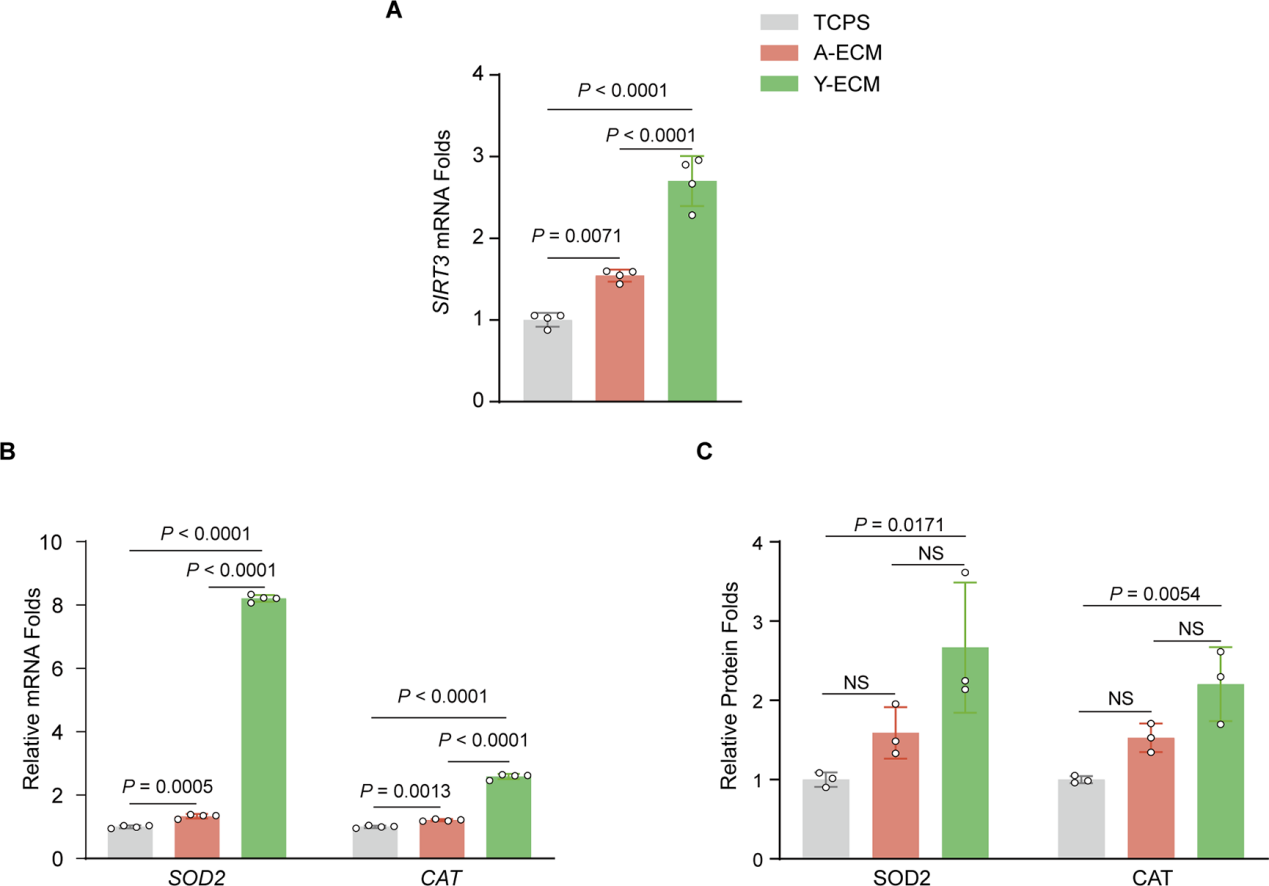


**Fig. S7. The effects of Y-ECM on the expression of antioxidant enzymes in A-BMMSCs.** A) Effects of different ECM interventions on *SIRT3* gene expression (n = 4 for each group, one-way ANOVA). B) Y-ECM up-regulated gene expression levels of antioxidant enzymes (*SOD2* and *CAT*) (n = 4 for each group, one-way ANOVA). C) Quantification of intracellular antioxidant enzymes protein levels (SOD2 and CAT) (n = 3 for each group, one-way ANOVA). Data are presented as mean ± SD of at least three independent assays for each experiment. Statistically significant differences between groups are set at *P* < 0.05.


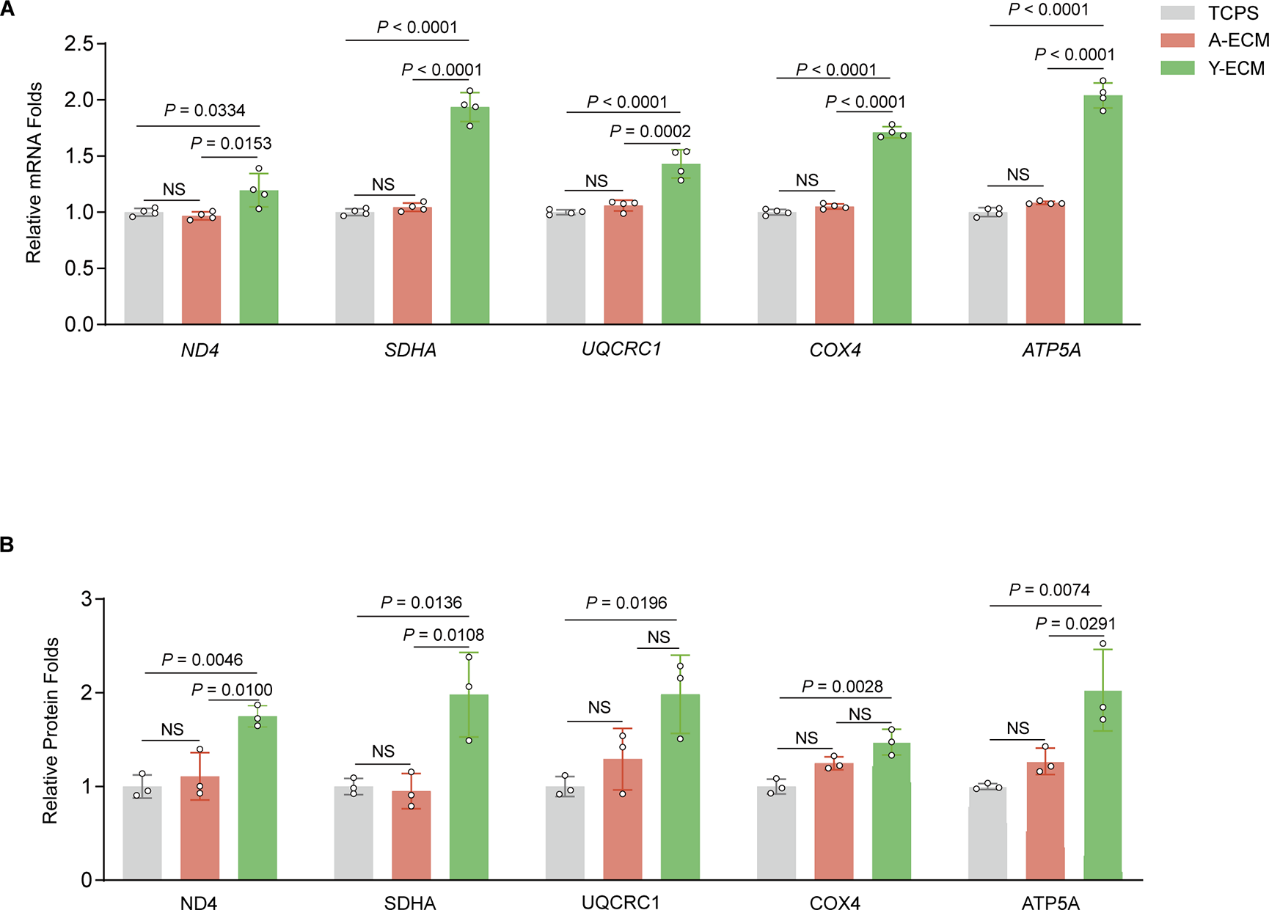


**Fig. S8. The effects of Y-ECM on mitochondrial respiratory chain factors in A-BMMSCs.** A) Y-ECM up-regulated gene expression levels of *ND4*, *SDHA*, *UQCRC1*, *COX4*, and *ATP5A* (n = 4 for each group, one-way ANOVA). B) Quantification of ND4, SDHA, UQCRC1, COX4, and ATP5A (n = 3 for each group, one-way ANOVA). Data are presented as mean ± SD of at least three independent assays for each experiment. Statistically significant differences between groups are set at *P* <0.05.

**
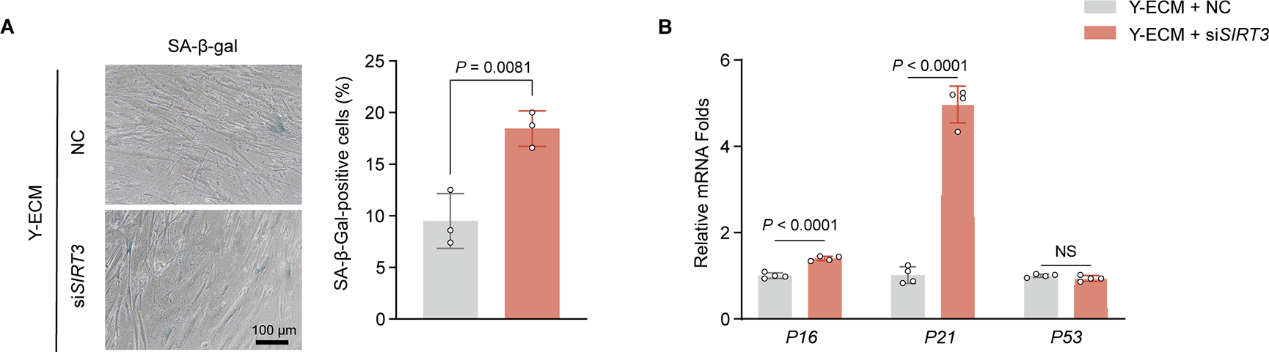
**

**Fig. S9. Silencing *SIRT3* reversed the Y-ECM-mediated anti-senescence effect.** A) SA-β-gal staining and quantification of SA-β-gal-positive cells (n = 3 for each group, unpaired two-tailed Student’s *t*-test). B) The effect of SIRT3 inhibition on gene expression of senescence-related genes (*P16*, *P21*, and *P53*) (n = 4 for each group, unpaired two-tailed Student’s *t*-test). Data are presented as mean ± SD of at least three independent assays for each experiment. Statistically significant differences between groups are set at *P* < 0.05.

**
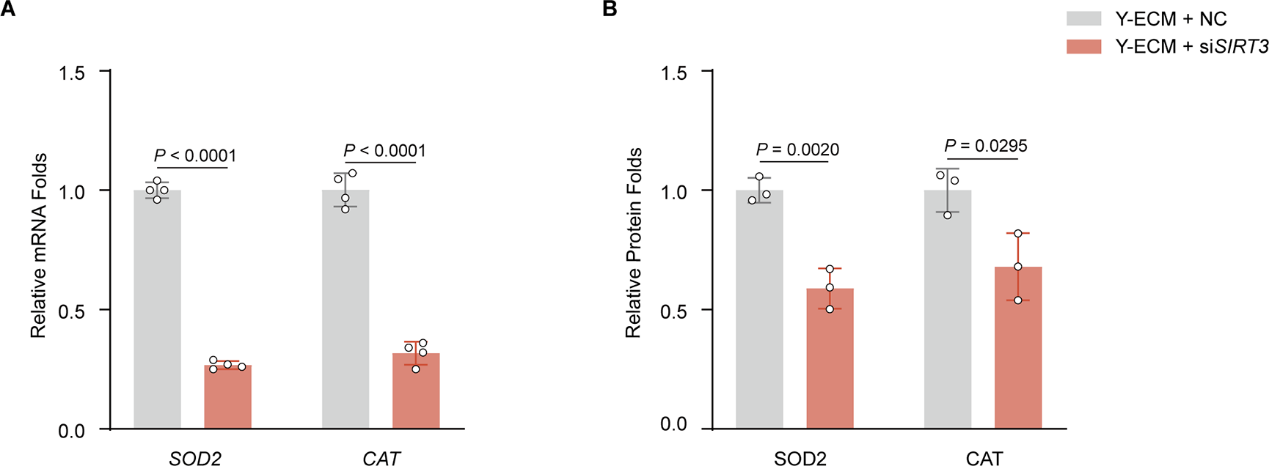
**

**Fig. S10. Silencing *SIRT3* abrogated the antioxidant effects of Y-ECM on A-BMMSCs**. A) Gene expression of antioxidant enzymes *SOD2* and *CAT* (n = 4 for each group, unpaired two-tailed Student’s *t*-test). B) Quantification of SOD2 and CAT protein levels (n = 3 for each group, unpaired two-tailed Student’s *t*-test). Data are presented as mean ± SD of at least three independent assays for each experiment. Statistically significant differences between groups are set at *P* < 0.05.


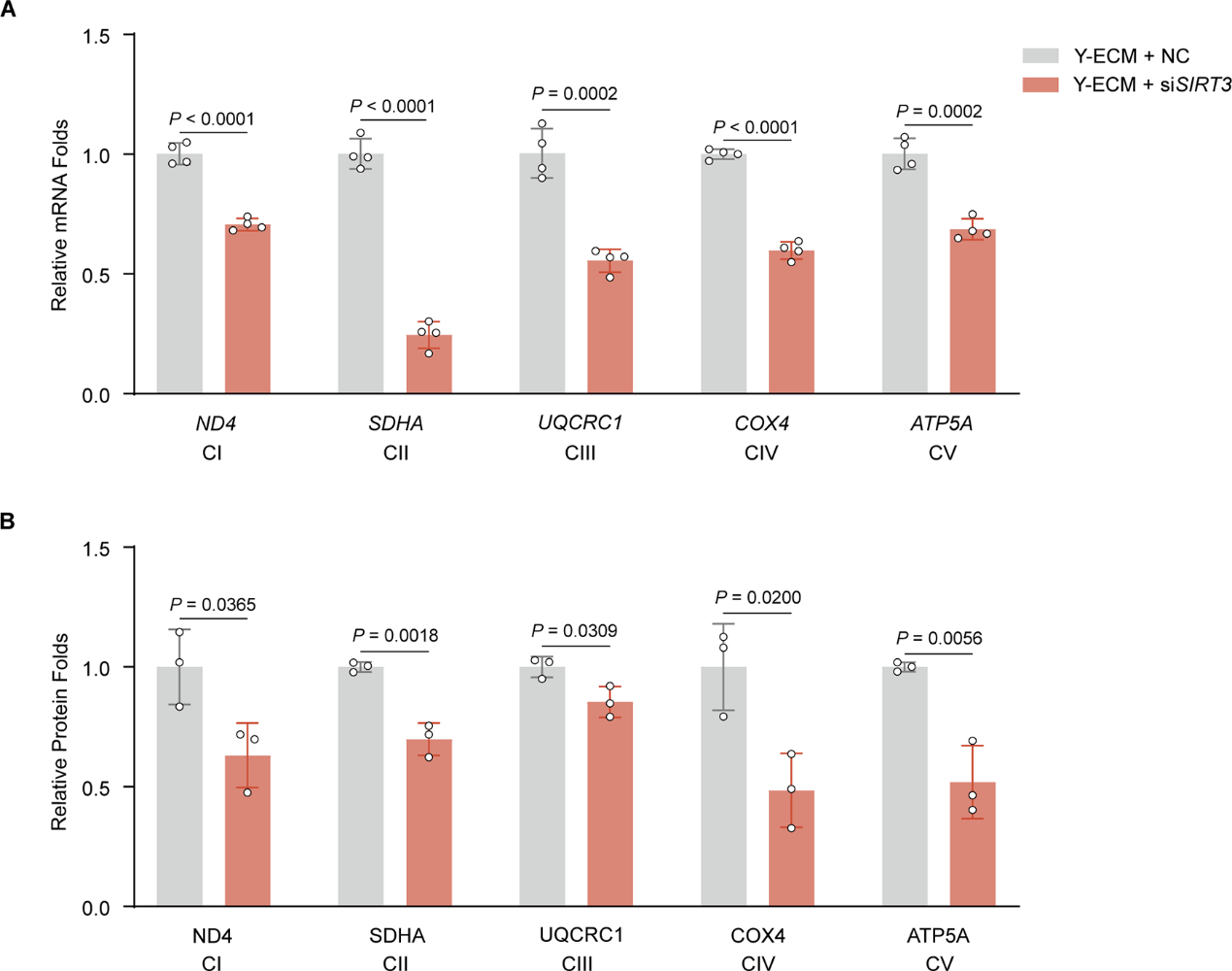


**Fig. S11. Silencing *SIRT3* abolished the protective effects of Y-ECM on mitochondrial functions**. A) The gene expression of mitochondrial respiratory chain factors (*ND4*, *SDHA*, *UQCRC1*, *COX4*, and *ATP5A*) (n = 4 for each group, unpaired two-tailed Student’s *t*-test). B) Quantification of the protein levels of ND4, SDHA, UQCRC1, COX4, and ATP5A (n = 3 for each group, unpaired two-tailed Student’s *t*-test). Data are presented as mean ± SD of at least three independent assays for each experiment. Statistically significant differences between groups are set at *P* < 0.05.


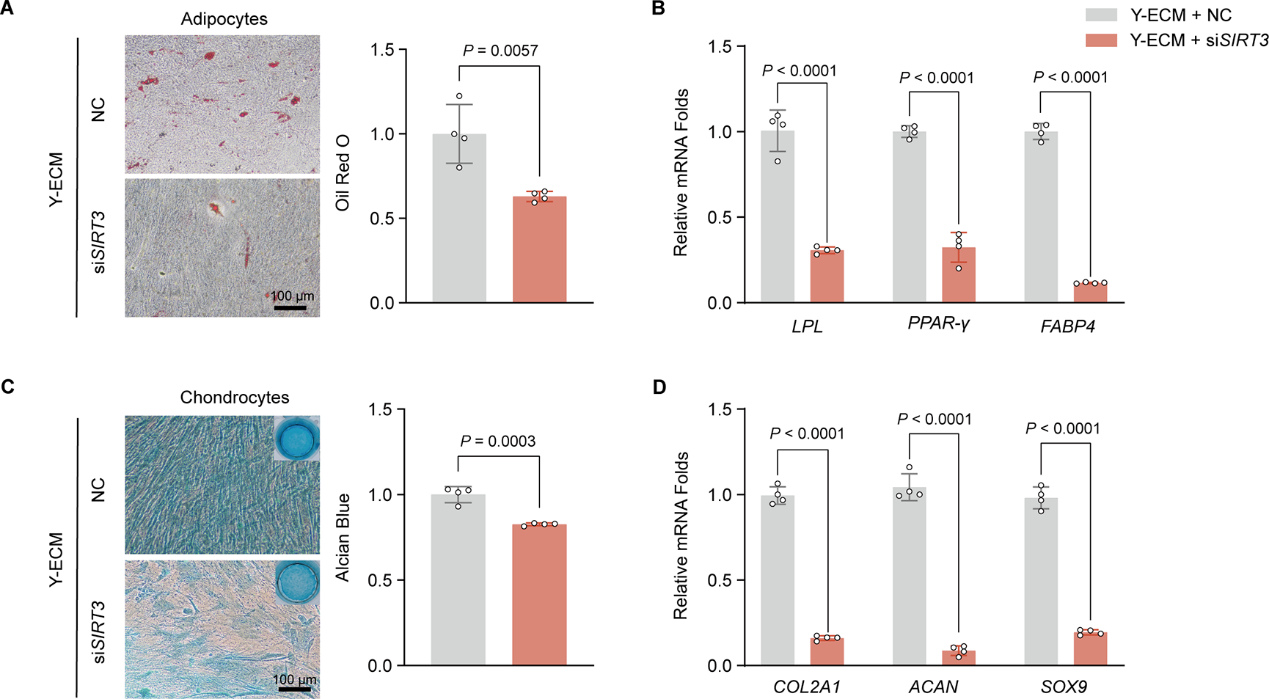


**Fig. S12. Silencing *SIRT3* abrogated the adipogenic and chondrogenic differentiation potentials of A-BMMSCs**. A) Representative images and quantitative analysis of lipid formation by Oil red O staining (n = 4 for each group, unpaired two-tailed Student’s *t*-test). B) The gene expression of adipocyte-specific genes (*LPL*, *PPAR-γ*, and *FABP4*) (n = 4 for each group, unpaired two-tailed Student’s *t*-test). C) Representative images and quantitative analysis of sulfated glycosaminoglycans (GAGs) by Alcian blue staining (n = 4 for each group, unpaired two-tailed Student’s *t*-test). D) The gene expression of chondrocyte-specific genes (*COL2A1*, *ACAN*, and *SOX9*) (n = 4 for each group, unpaired two-tailed Student’s *t*-test). Data are presented as mean ± SD of at least four independent assays for each experiment. Statistically significant differences between groups are set at *P* < 0.05.

**
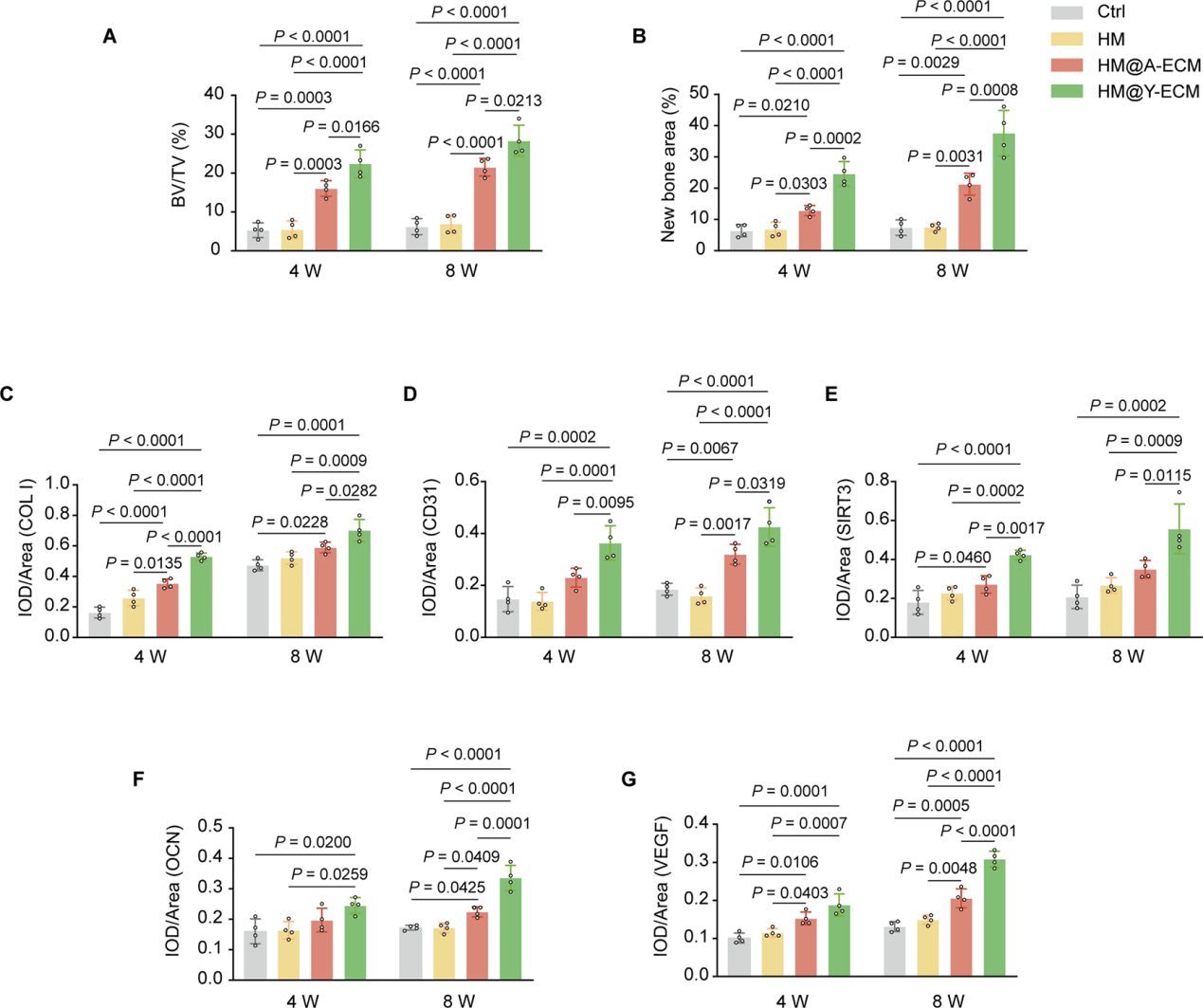
**

**Fig. S13. HM@Y-ECM promoted vascularized bone regeneration in aged rats**. A) BV/TV of the newly formed bone after 4 and 8 weeks of treatment (n = 4 for each group, one-way ANOVA). B) Quantitative analysis of the new bone area at 4 and 8 weeks (n = 4 for each group, one-way ANOVA). C-E) Quantitative analysis for C) COL I, D) CD31, E) SIRT3, F) OCN, and G) VEGF expression at 4 and 8 weeks (n = 4 for each group, one-way ANOVA). Data are presented as mean ± SD of at least four independent assays for each experiment. Statistically significant differences between groups are set at *P* < 0.05.

**Table S1: The primer sequences used for quantitative real-time RT-PCR.**

| **Gene** | **Forward Primer sequence (5’-3’)** | **Reverse Primer sequence (5’-3’)** |
| --- | --- | --- |
| *GAPDH* | AGAAAAACCTGCCAAATATGATGAC | TGGGTGTCGCTGTTGAAGTC |
| *ND4* | TGGCTATCATCACCCGATG | TGAGTAGTAGAATGTTTAGTGAGC |
| *SDHA* | TTTATCAGCGTGCATTTGGT | ACGTGCAGCTGAAGTAGGTG |
| *UQCRC1* | AATGTCAGGAAGCTGTCTCGT | TATGTCCATGGGATGCCACC |
| *COX4* | ATGTCAAGCACCTGTCTGC | CCCTGTTCATCTCAGCAAA |
| *ATP5A* | CTCGCTTCGTTGCCACTTC | TAGGCGGCTCTATCTCTCGT |
| *ND1* | CATTCCTAATGCTTACCGAACGA | TGAGAGCTAAGGTCGGGGC |
| *β-GLOBIN* | GTGCTCGGTGCCTTTAGTGA | GGGGAAAGAAAACATCAAGCGT |
| *SOD2* | GGGGATTGATGTGTGGGAGCACG | AGACAGGACGTTATCTTGCTGGGA |
| *CAT* | TGGGATCTCGTTGGAAATAACAC | TCAGGACGTAGGCTCCAGAAG |
| *SIRT1* | GCGGGAATCCAAAGGATAAT | CTGTTGCAAAGGAACCATGA |
| *SIRT2* | TGCTGATGAGGGAGGCAAAGG | TCTGAGGTGACGCCCAAGTGT |
| *SIRT3* | ACAGCAACCTCCAGCAGTACGA | CGTGTAGAGCCGCAGAAGCA |
| *SIRT4* | AGGTCAGAAAAAGTGGGGCTT | CCTACGAAGTTTCTCGCCCA |
| *SIRT5* | CTTGTGGAGTTGTGGCTGAGA | CCTCTTCACACCCTTTTCCTGA |
| *SIRT6* | GCAGTCTTCCAGTGTGGTGT | AAGGTGGTGTCGAACTTGGG |
| *SIRT7* | CAGGGAGTACGTGCGGGTGT | TCGGTCGCCGCTTCCCAGTT |
| *P16* | GTGCTCACTCCAGAAAACTC | AATGTCCTGCCTTTTAACGTAG |
| *P21* | CTGTGATGCGCTAATGGCG | AAGTCGAAGTTCCATCGCTCA |
| *P53* | ATCTACAAGCAGTCACAGCAC | TTCCTTCCACTCGGATAAGATGC |
| *ACAN* | GTGCCTATCAGGACAAAGGTCT | GATGCCTTTCACCACGACTTTC |
| *COL2A1* | GAGACAGCATGACGCCGAG | GCGGATGCTCTCAATCTGGT |
| *SOX9* | GGCAAGCTCTGGAGACTTCTG | CTGCAGCGCCTTGAAGATG |
| *RUNX2* | AGAAGGCACAGACAGAAGCTTGA | AGGAATGCGCCCTAAATCACT |
| *COL1A1* | CAGCCGCTTCACCTACAGC | TTTTGTATTCAATCACTGTCTTGCC |
| *ALP* | AGCACTCCCACTTCATCTGGAA | GAGACCCAATAGGTAGTCCACATTG |
| *PPAR-γ* | CCATCGAGGACATCCAAGACAACC | GTGCTCTGTGACAATCTGCCTGAG |
| *LPL* | CTGGACGGTAACAGGAATGTATGAG | CATCAGGAGAAAGACGACTCGG |
| *FABP4* | AACCTTAGATGGGGGTGTCCTG | TCGTGGAAGTGACGCCTTTC |

**Table S2: The information of primary anti-body.**

| Anti-body | Concentration | Supplier | Catalogue Number |
| --- | --- | --- | --- |
| For Western blot | | | |
| ND4 | 1:1000 | Abclonal | A9941 |
| SDHA | 1:2000 | Abclonal | A13852 |
| UQCRC1 | 1:2000 | Abclonal | A3339 |
| COX4 | 1:2000 | Abclonal | A11631 |
| ATP5A | 1:1000 | Abclonal | A11217 |
| SIRT3 | 1:1000 | Abclonal | A20805 |
| CAT | 1:2000 | Abclonal | A11220 |
| SOD2 | 1:2000 | Abclonal | A1340 |
| β-actin | 1:20000 | Abclonal | AC026 |
| For Immunofluorescent and immunohistochemical staining | | | |
| ATP5A | 1:100 | Abclonal | A11217 |
| COL I | 1:200 | Abclonal | A1352 |
| OCN | 1:200 | Proteintech | 23418-1-AP |
| VEGF | 1:200 | ImmunoWay | YN5444 |
| CD31 | 1:500 | Abclonal | A19014 |
| SIRT3 | 1:100 | Abclonal | A20805 |
